# circPTEN1, a circular RNA generated from *PTEN*, suppresses cancer progression through inhibition of TGF-β/Smad signaling

**DOI:** 10.1186/s12943-022-01495-y

**Published:** 2022-02-08

**Authors:** Lin Zheng, Hui Liang, Qiaoling Zhang, Zichu Shen, Yixin Sun, Xuyang Zhao, Jingjing Gong, Zhiyuan Hou, Kewei Jiang, Quan Wang, Yan Jin, Yuxin Yin

**Affiliations:** 1 Institute of Systems Biomedicine, Department of Pathology, School of Basic Medical Sciences, Beijing Key Laboratory of Tumor Systems Biology, Peking-Tsinghua Center of Life Sciences, Peking University Health Science Center, Beijing, 100191 China; 2 Institute of Precision Medicine, Peking University Shenzhen Hospital, Shenzhen, 518036 China; 3 Department of Gastroenterological Surgery, Laboratory of Surgical Oncology, Beijing Key Laboratory of Colorectal Cancer Diagnosis and Treatment Research, Peking University People’s Hospital, Beijing, 100044 China

**Keywords:** PTEN, circRNA, TGF-β/Smad signaling, Colorectal cancer, Tumor metastasis

## Abstract

**Background:**

*PTEN* is one of the most frequently mutated genes in human cancer. Although the roles of canonical PTEN protein and PTEN isoforms have been extensively explored, the current understanding of PTEN family members cannot fully illustrate the diversity of their roles in biological processes and tumor development. Notably, the function of noncoding RNAs arising from *PTEN* has been less elucidated.

**Methods:**

We searched circBase and circInteractome to analyze the potential of *PTEN* for generating circRNAs. Then, Sanger sequencing, RNase R and Actinomycin D assays were used to verify the ring structure of circPTEN1. In situ hybridization and qRT-PCR were used to determine the level of circPTEN1 in peritumor and tumor tissues of colorectal cancer (CRC). Furthermore, functional experiments, including Transwell assay, 3D multicellular tumor spheroid invasion assay and metastasis models, were performed using circPTEN1 knockdown and overexpression cell lines in vitro and in vivo to investigate the effects of circPTEN1 on tumor metastasis in CRC. Mechanistically, luciferase reporter assay, fluorescence in situ hybridization, electrophoretic mobility shift assay, RNA immunoprecipitation, RNA pull-down and mass spectrometry were executed.

**Results:**

We identified a circular RNA generated from the *PTEN* gene, designated circPTEN1, that is frequently downregulated in colorectal cancer, and decreased expression of circPTEN1 predicts poor survival. Low expression of circPTEN1 promotes metastasis in PDX models in vivo and accelerates cancer cell invasion in vitro, whereas overexpression of circPTEN1 reveals opposite roles. Mechanically, we found that circPTEN1 is capable of binding the MH2 domain of Smad4 to disrupt its physical interaction with Smad2/3, which reduces the formation and subsequent nucleus translocation of Smad complexes and consequently suppresses the expression of its downstream genes associated with epithelial-mesenchymal transition upon TGF-β stimulation. Furthermore, we found that eIF4A3 suppresses the cyclization of circPTEN1 by directly binding to the circPTEN1 flanking region.

**Conclusions:**

Our study uncovered a novel *PTEN* gene-generated circRNA with a tumor suppression function, and further revealed the mechanism of circPTEN1 in CRC metastasis mediated by TGF-β. The identification of circPTEN1 provides a new direction for *PTEN* investigation, and elucidation of circPTEN1/TGF-β/Smad signaling may pave the way for the development of a potential therapeutic strategy for the suppression of cancer progression.

**Supplementary Information:**

The online version contains supplementary material available at 10.1186/s12943-022-01495-y.

## Background

Phosphatase and tensin homolog (*PTEN*), which is located on chromosome 10q23 and encodes a 403-amino acid protein that has both phosphatase-dependent and phosphatase-independent functions, is one of the most frequently mutated tumor suppressor genes in human cancer [1]. *PTEN* acts as a haploinsufficient tumor suppressor gene, and genetic ablation of *PTEN* accelerates the progression of multiple human cancers [2]. Beyond tumor suppression, *PTEN* has a key role in a variety of biological processes, including cell metabolism, cell motility, genome maintenance and cellular senescence [1]. The best characterized function of the canonical PTEN protein is the ability to dephosphorylate PtdIns (3, 4, 5) P3 and convert it back into PIP2 in the cytoplasm, therefore antagonizing the PI3K/AKT pathway, which is mainly involved in the regulation of cell growth, differentiation, proliferation and invasion [2]. More recently, three conserved PTEN isoforms, PTENα (also known as PTEN-Long), PTENβ and PTENε, were sequentially identified to be initiated from in-frame non-AUG codons in the 5′ untranslated region (5’UTR) of PTEN mRNA [3–6]. PTENα/β/ε are demonstrated to be involved in distinct functional biological processes, such as mitophagy and neutrophil chemotaxis [7], through interaction with diverse proteins with the N-terminal extended domain. Moreover, the results from different studies indicate that these PTEN isoforms show tumor suppressive or promoting roles with distinct mechanisms, which further highlights the complexity of the role *PTEN* plays in tumor development [6, 8]. The study of PTENα/β/ε advances our understanding of the functions of *PTEN*. Nonetheless, the current exploration of the canonical PTEN protein and PTEN isoforms cannot fully illustrate the diversity of their involvement in biological processes and tumor development. Notably, the function of noncoding RNAs arising from *PTEN* has been less elucidated.

Circular RNAs (circRNAs) are a class of single-stranded, covalently closed RNA molecules that are formed by precursor mRNA back-splicing or skipping events of thousands of genes in eukaryotes [9]. Advances in RNA sequencing and bioinformatics tools have boosted the discovery of various circRNAs and uncovered their important functions [10]. Functionally characterized circRNAs have critical roles in gene regulation through various actions, including sponging microRNAs and proteins as well as regulating transcription and splicing [11]. Compelling experimental evidence shows that circRNAs are widely involved in the initiation and progression of human cancers by participating in the regulation of one or several cancer hallmarks, including the activation of invasion and metastasis, and the expression of circRNAs is frequently deregulated during cancer onset, progression, and dissemination [12]. Moreover, an increasing number of studies have demonstrated that circRNA-based diagnostic and therapeutic strategies show great potential in cancer management [13]. However, unlike miRNAs, the expression, role and molecular mechanism of most circRNAs in regulating cancer progression remain largely unknown. Regarding the important roles of circRNAs in tumorigenesis, the discovery and functional exploration of circRNAs is urgent.

Colorectal cancer (CRC) is one of the most frequent cancers worldwide among both females and males. The development of CRC is a multistep process involving various factors, such as genetic mutations. Despite progress in the diagnosis and therapeutic improvements of this disease, the prognosis of CRC patients remains poor. Clinically, metastasis is the overwhelming cause of death in patients with CRC, and the liver is the most frequent distant metastatic site [14]. Due to distant tumor metastasis, the 5-year survival rate of patients with advanced stages of CRC is only 12% [15]. This poor long-term survival outlook highlights that exploring the pathogenesis mechanisms of CRC metastasis is urgent and essential for treating CRC patients. Although previous studies have demonstrated that circular RNA abnormalities are highly involved in the pathogenic process of metastasis of CRC [16], the precise molecular mechanisms remain largely unclear.

In the current study, we identified one circRNA derived from the *PTEN* gene (circPTEN1) as a suppressor of CRC progression. We verified that circPTEN1 is significantly downregulated in CRC and that its expression is positively correlated with patient prognosis. We further demonstrated that circPTEN1 inhibits CRC metastasis both in vitro and in vivo. Mechanistically, circPTEN1 binds to the MH2 domain of Smad4 to disrupt the physical interaction between Smad4 and Smad2/3, leading to suppression of Smad complex nucleus translocation and subsequent transcriptional activation of downstream genes associated with EMT activation under TGF-β stimulation. Thus, circPTEN1 might be a potential therapeutic target for CRC prevention. Identification and functional exploration of circRNAs arising from *PTEN* advance our understanding of the diversity and complexity of *PTEN* functions in physiological and pathological processes.

## Methods

### Cell lines, antibodies, and reagents

All human cell lines used in this study were from the American Type Culture Collection. These cell lines were authenticated by STR locus analysis and tested for mycoplasma contamination. SW480 cells were maintained in RPMI 1640 (CORNING), and all other cells were maintained in DMEM (CORNING), supplemented with 10% FBS (HyClone) in a 37 °C incubator with 5% (v/v) CO_2_. Primary antibodies used for western blot, immunoprecipitation, RIP and immunofluorescence are listed in Supplementary Table 1.

### Human tissue samples

CRC and adjacent nontumorous tissue samples were freshly resected from 150 patients at the People’s Hospital of Peking University from September 2018 to December 2020. All samples were verified by diagnostic pathology and stored in liquid nitrogen for RNA extraction.

### Plasmids and cloning strategies

The plasmids pCMV-tag-2b, pLKO.1-puro, pEGFP-N1 and pLV-puro were purchased from Addgene. Smad2, Smad3 or Smad4 and mutations of these molecules were inserted into these plasmids. The pLCDH-ciR plasmid was purchased from Geneseed (China).

### CircRNA expression vectors

The circPTEN1 transcript in which the sh-circPTEN1#2 targeted sequence was mutated (circPTEN1-RS) was PCR amplified using primers F (5′- AATCTGTTCAATTAACGAATTCTGAAATATGCTATCTTACAGCCACAGGCTCCCAGACATG-3′) and R (5′-ATCATCCCAAATTAGTGGATCCTCAAGAAAAAATATATTCACCTTTTTGTCTCTGGTCCTTACTTCCC-3′), digested by EcoRI and BamHI, and ligated into pLCDH-ciR to create circPTEN1-RS-overexpressing plasmids. The expression level of this construct was detected by northern blot.

### Cytosolic/nuclear fractionation

Cells were resuspended in hypotonic buffer (25 mM Tris-HCl, pH 7.4, 1 mM MgCl_2_, 2.5 mM KCl) and incubated on ice for 5 min before adding an equal volume of hypotonic buffer containing 1% NP-40 for another 5 min. After centrifuging the cells at 5000 g for 5 min, the supernatant was collected as the cytosolic fraction. The pellets were washed twice with hypotonic buffer and then resuspended in nuclear resuspension buffer (20 mM HEPES, pH 7.9, 400 mM NaCl, 1 mM EDTA, 1 mM EGTA, 1 mM DTT, 1 mM PMSF). After incubation on ice for 30 min, the sample was centrifuged at 12,000 g for 10 min, and the supernatant was collected as the nuclear fraction. β-actin and U1 served as controls.

### Co-immunoprecipitation

Cells were extracted and lysed in lysis buffer (50 mM Tris-HCl, pH 7.5, 150 mM NaCl, 0.5% NP-40) freshly supplemented with protease inhibitors. One microgram of Smad4 antibody was incubated with 100 μl of protein A/G beads in lysis buffer for 2 h. After washing twice with lysis buffer, the bead-antibody complex mixture was sequentially incubated with cell lysates containing 800 μg of protein. The protein-bead complex mixture was washed in washing buffer containing 0.1% NP-40 and subjected to western blotting to evaluate protein interactions.

### RNA immunoprecipitation (RIP) assays

The association of Smad4 with circPTEN1, and eIF4A3 with circPTEN1 upstream sequences were analyzed by RIP. Briefly, cells were lysed in RIP lysis buffer (20 mM Tris-HCl, pH 7.5, 100 mM KCl, 5 mM MgCl_2_ and 0.5% NP-40) supplemented with RNase and protease inhibitors. The lysates were incubated with eIF4A3, Smad4 or control IgG antibody for 4 h at 4 °C followed by incubation with protein-A/G Dynabeads for 2 h at 4 °C with rotation. After three washes with 500 μl washing buffer (50 mM Tris-HCl, pH 7.4, 150/300/600 mM NaCl, 0.05% Tween-20), RNA from the input (5%) and from the immunoprecipitated fractions was extracted by TRIzol (Ambion), followed by RQ1 DNase (Promega) digestion and ethanol precipitation. The RNA was used for qRT-PCR analysis, and the primers used are listed in supplementary Table 2.

### RNA fluorescence in situ hybridization (FISH) and protein immunofluorescence (IF) 

RNA FISH assays were performed as previously described [17]. Briefly, complementary probes targeting the “head-to-tail” sequence of circPTEN1 were synthesized by Sangon Biotech (Shanghai, China) (Table S2). The cells were cultured on gelatin-coated glass cover slips, fixed with 4% formaldehyde in phosphate-buffered saline (PBS), and permeabilized for 5 min with 0.2% Triton X-100 in PBS. The cover slips were incubated overnight at 37 °C with a hybridization mix containing 1 ng/μL probe mix. The unbound probes were washed off the next day with hybridization buffer, and cells were mounted after staining with DAPI. Images were taken with an immunofluorescence microscope (Nikon A1).

For protein IF assays, cells seeded on cover glass were fixed with 4% PFA for 15 min and washed twice with PBS. Washed cells were permeabilized with 0.2% Triton X-100 for 5 min and blocked for 1 h using 2% BSA dissolved in PBS. The primary antibody was then applied for 3 h at room temperature, followed by three washes in PBS, and fluorophore-conjugated secondary antibody was applied for 1 h. After being washed twice with PBS, cells were stained with DAPI (Sigma). Coverslips were mounted, and cells were evaluated with fluorescence microscopy. A Nikon A1 microscope was used for confocal microscopy.

### In vitro transcription

The DNA template used for the in vitro synthesis of biotinylated circPTEN1 or the flaking sequences of the circPTEN1 transcript was generated by PCR. As shown in Table S2, the forward primer contained the T7 RNA polymerase promoter sequence to allow for subsequent in vitro transcription. PCR products were purified using the DNA Gel Extraction Kit (GENERAY BIOTECH), and in vitro transcription was performed using the T7-Flash Biotin RNA Transcription Kit (Epicenter, biotin labeling) or Transcript Aid T7 High Yield Transcription Kit (Thermo Scientific, without biotin). RNA was purified by phenol-chloroform extraction and then used for in vitro cyclization or biotin RNA pull-down.

### In vitro cyclization

In vitro cyclization of linear RNA was performed as previously described [18]. Briefly, biotin-labeled or unlabeled RNA obtained by in vitro transcription was incubated with the indicated DNA splints (molar ratio = 1:1.5) at 90 °C for 5 min, and then cooled to room temperature over 20 min. Ligation to form circRNAs was then performed overnight at 16 °C with T4 DNA ligase (NEB), and then the sample was treated with RNase R and DNase I at 37 °C for 30 min. RNA was subsequently purified by phenol-chloroform extraction. DNA splint sequence: TCTGGGAGCCTGTGGCTTTTTGTCTCTGGT.

### Biotin RNA pull-down assay

Cells were lysed by ultrasonication in RIP buffer (150 mM KCl, 25 mM Tris-HCl, pH 7.4, 0.5 mM dithiothreitol, 0.5% NP-40) supplemented with protease inhibitors and RNase inhibitors, and the cell lysates were then precleared with streptavidin magnetic beads (Invitrogen). In vitro transcribed biotin-labeled RNA or DNA probes adsorbed to streptavidin magnetic beads were then incubated with cell lysates at 4 °C for 6 h before washing three times with RIP buffer. Afterwards, the potential interacting proteins were evaluated with western blot or mass spectrometry analysis.

### Northern blot

Northern blotting was performed with the DIG Northern Starter Kit (Roche) according to the manufacturer’s instructions with minor modifications. Briefly, the DNA template used for the in vitro synthesis of probes labeled with digoxigenin to detect circPTEN1 or linear PTEN mRNA was generated by PCR, and the primers are listed in Table S2. Ten milligrams of total RNA with or without RNase R digestion was resolved on 2% agarose gels prepared with formaldehyde before transfer to a Hybond-N+ membrane (Solarbio) by capillary transfer, and RNA was then fixed to the membrane through UV crosslinking (200,000 mJ/cm^2^ at 265 nm). Hybridization was performed at 68 °C for 6 h with a biotin-labeled oligonucleotide probe. The membranes were blocked in blocking buffer for 30 min, and then incubated with antibody solution for 30 min with gentle shaking. The membranes were washed three times with washing buffer, incubated with detection solution for 5 min and exposed to X-ray film. GAPDH was used as an internal control.

### Recombinant protein purification

Flag-tagged Smad4, Smad4-MH1 and Smad4-MH2 expressed in 293 T cells were purified using ANTI-FLAG M2 affinity gel (Sigma) according to the manufacturer’s instructions. The immunoprecipitates were then eluted with 3xFlag peptides.

### Electrophoretic mobility shift assays

EMSAs were performed using the Light Shift Chemiluminescent RNA EMSA Kit according to the manufacturer’s protocol (Thermo Scientific). Briefly, biotin-labeled in vitro cyclized circPTEN1 (BL circPTEN1, 2 nM) was incubated with purified Flag-Smad4, Smad4-MH1 or Smad4-MH2 proteins. Unlabeled cyclized circPTEN1 (10 mM) was used as a competitor. Reactions were subjected to PAGE using native gels, and after transfer to nylon membranes, the biotin-labeled RNA was detected using SA-HRP and ECL.

### 3D multicellular tumor spheroids invasion assay

Plate 200 μL of cell suspension (2 × 10^4^ cells/well) in 96-well ULA round bottom dishes, and incubate dishes using standard culture conditions for 72 h to generate multicellular tumor spheroids (MTSs). The MTSs were then transferred to 96-well ULA round bottom dishes coated with 2% type I collagen. After transferring all MTSs to the coated wells, the hydrogel was allowed to polymerize at 37 °C for 20 min and then overlaid with 200 μl standard culture medium. Images were captured and saved for each MTS using an inverted microscope (t = 0 day). The medium was exchanged every second day for 1 week, and field images were acquired every day. The quantification of the invading area was analyzed using Olympus cellSens Standard 1.9.

### Transwell migration and invasion assay

Transwell migration assays were performed in a 24-well Boyden chamber (6.5 mm diameter, 8.0 μm; BD) according to the manufacturer’s instructions as previously described [3]. In brief, 5 × 10^4^ cells/well in serum-free medium were seeded in the upper chamber, and the lower chamber was filled with complete media. After 24 h, cells in the upper chamber were gently removed. Migrated cells on the lower side of the membrane were fixed with methanol, stained with 0.1% crystal violet, and then counted at 40× magnification in five random fields per well. For the in vitro invasion assay, experiments were performed with Matrigel matrix-coated Transwell chambers.

### Animal models

The BALB/c nude mice used in this study were obtained from Vital River Laboratory (Beijing, China). For the liver metastasis model, 2 × 10^6^ cells suspended in 40 μl PBS were injected into the inferior hemispleen of 6-week-old BALB/c nude mice. For the lung metastasis model, 5 × 10^5^ cells suspended in 100 μl PBS were injected travenously in the tail vein into each 4-week-old BALB/c nude mouse. For the LM model, tumors were imaged 8 weeks after cell injection, and for the lung metastasis model, tumors were imaged 6 weeks after cell injection. Briefly, VivoGlo™ Luciferin (Promega, Madison, WI) was dissolved in sterilized PBS (final concentration: 15 mg/ml). Mice were anesthetized with isoflurane and injected traperitoneally with 100 μl of the luciferin solution. After 15 min, images were acquired with the Xenogen IVIS Lumina series II for 5 min and analyzed using the Living Image 2.11 software package (Xenogen Corp.).

### Statistical analysis

Prism GraphPad software v5.0 was used for analysis. Each experiment was performed in triplicate, and the data are shown as the mean ± S.D., unless otherwise stated. Student’s two tailed paired t test was used to determine the statistical significance of differences in the values representing the expression of circPTEN1 or eIF4A3 between CRC tissues and corresponding normal tissues, and Student’s two tailed unpaired t test was used to determine the statistical significance of the other in vitro experiments. Associations of circPTEN1 expression with clinicopathological characteristics were analyzed by chi-square Fisher’s exact tests. Associations of circPTEN1 expression with the prognosis of CRC patients were analyzed by Kaplan-Meier analysis with the log-rank test. Correlations of circPTEN1 expression and eIF4A3 expression were analyzed using the Pearson correlation test. *P* values of 0.05 or less were considered significant.

## Results

### Identification analysis of a novel circular RNA generated from the *PTEN* gene

**Fig. 1:**
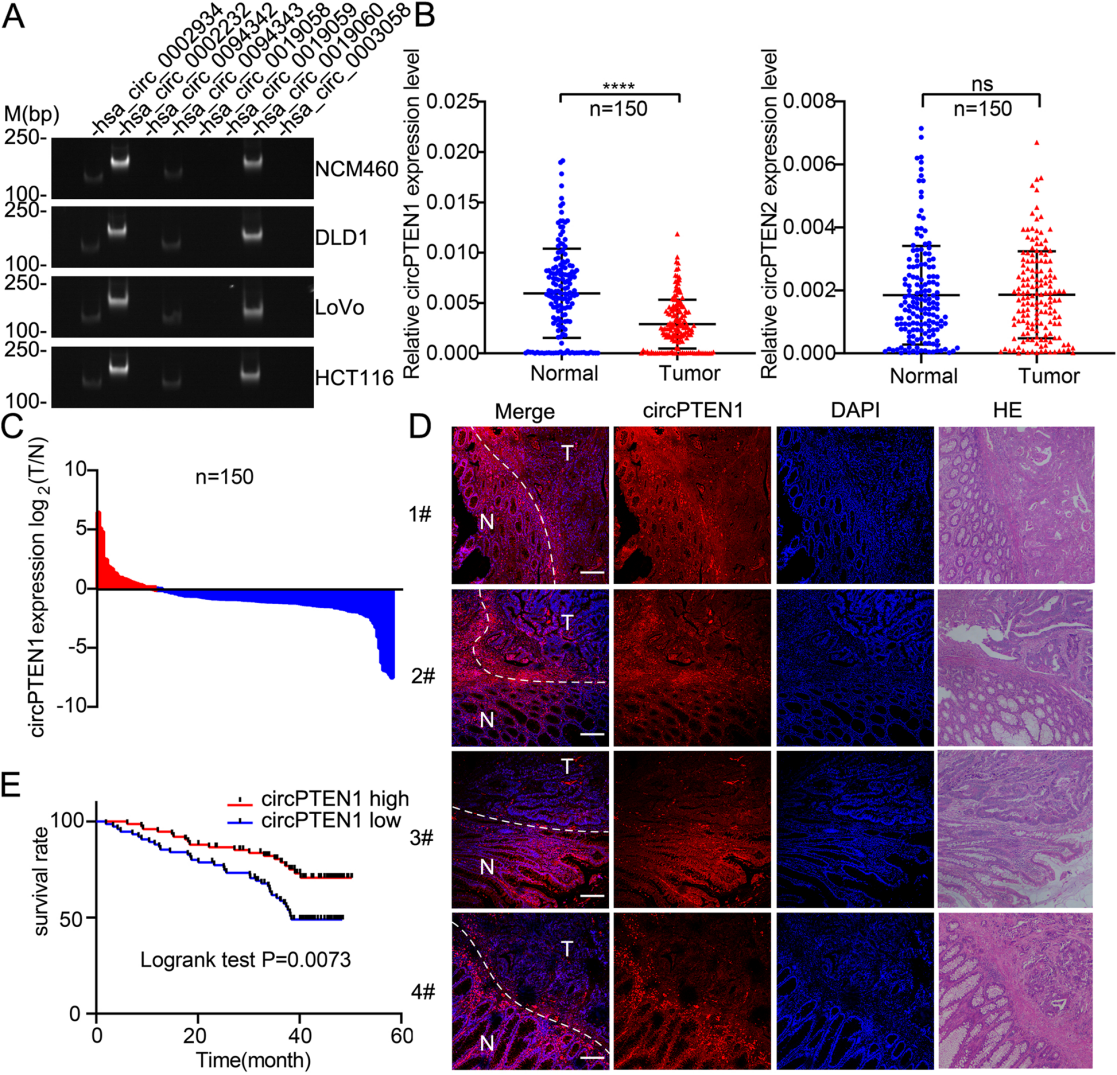
Identification analysis of a novel circular RNA generated from the *PTEN* gene. **A**. The expression profiles of circRNAs derived from the *PTEN* gene in the normal human colonic epithelial cell line NCM460 and colon cancer cell lines. **B**. qRT-PCR assay showing the relative levels of circPTEN1 and circPTEN2 (normalized to β-actin) in the peritumor and tumor tissues of colon cancer (*n* = 150). ****, *P* < 0.0001. ns: no statistically significance. **C.** circPTEN1 expression was significantly lower in tumor tissues than in peritumor tissues in 80% of colon cancer patients. **D.** Fluorescence in situ hybridization assay was conducted to determine the expression of circPTEN1 in the peritumor and tumor tissues of colon cancer. N: peritumor tissue, T: tumor tissue. The scale bars represent 200 μm. **E.** Kaplan-Meier analysis of correlations between circPTEN1 expression levels and OS (overall survival) of 150 colon cancer patients

We sought to uncover circRNAs arising from the *PTEN* gene and explore their involvement in CRC development. Firstly, we analyzed the potential of *PTEN* for generating circRNAs with online circRNA databases. Mining of circBase [19] and circInteractome [20] revealed 8 potential circRNAs derived from *PTEN* (Table 1). Based on the predicted sequences from these databases, we designed specific divergent primers that were predicted to amplify only the circular transcript. Further validation by RT-PCR with divergent primers revealed that hsa_circ_0002232 and hsa_circ_0019060 were more highly expressed in the normal human colonic epithelial cell line NCM460 and colon cancer cell lines DLD1, LoVo and HCT116 relative to other circRNAs derived from *PTEN* (Fig. 1A). Given that hsa_circ_0002232 and hsa_circ_0019060 are derived from the host gene *PTEN*, we termed them circPTEN1 and circPTEN2, respectively. We next measured the expression of circPTEN1 and circPTEN2 in CRC patients by qRT-PCR in matched sets of CRC tissues and corresponding normal tissues adjacent to the tumor (NATs), and a cohort of 150 CRC patients with survival data was included. The data showed that the expression of circPTEN1, but not circPTEN2, was significantly downregulated in CRC tissues compared with the corresponding NATs (Fig. 1B). Additionally, circPTEN1 expression was lower in CRC tissues in 80% of patients (Fig. 1C). FISH analysis further confirmed that circPTEN1 was prominently expressed at lower levels in CRC tissues than in adjacent normal tissues (Fig. 1D and Supplementary Fig. 1). Therefore, we focused on circPTEN1, and subsequently investigated its clinicopathological significance in CRC patients. According to circPTEN1 expression, colon cancer patients were classified into high- and low-level groups. Notably, circPTEN1 levels in patients with lymph node metastases (N1 + N2), stage III–IV or distant metastases were lower than those in patients without lymph node metastases (N0), stage I–II or nonmetastases, while no significant correlation was observed concerning other clinicopathological features, including age, sex, tumor size and differentiation (Table 2). Since lymph node metastasis (LM) is an important prognostic prediction factor in patients with liver metastasis, which is the leading cause of CRC mortality, the close correlation of circPTEN1 level with LM indicates that it may be involved in the regulation of CRC progression. Furthermore, from Kaplan-Meier analysis, we found that patients with lower circPTEN1 expression had a significantly shorter overall survival than patients with higher circPTEN1 expression (Fig. 1E). These findings indicated that circPTEN1 may serve as a tumor suppressive noncoding RNA and that circPTEN1 dysregulation may contribute to CRC development and progression.

**Table 1:** CircRNAs arised from *PTEN* as revealed by circBase and circInteractome

Circular RNA ID	Location	Genomic Length (bp)	Spliced Length (bp)	RNAseq resources
hsa_circ_0002934	chr10:89685269–89,693,008	7739	328	Jeck2013 (PMID:23249747)
hsa_circ_0002232	chr10:89624210–89,693,008	68,798	508	Jeck2013 (PMID:23249747)
hsa_circ_0094342	chr10:89653781–89,655,534	1753	1753	Salzman2013 (PMID:24039610)
hsa_circ_0094343	chr10:89726897–89,727,085	188	188	Rybak2015 (PMID:25921068)
hsa_circ_0019058	chr10:89653781–89,653,866	85	85	Rybak2015 (PMID:25921068)
hsa_circ_0019059	chr10:89690802–89,712,016	21,214	425	Jeck2013 (PMID:23249747)
hsa_circ_0019060	chr10:89690802–89,717,776	26,974	592	Salzman2013 (PMID:24039610)
hsa_circ_0003058	chr10:89635161–89,638,780	3619	3619	Salzman2013 (PMID:24039610)

circRNAs that are generated by back splicing of *PTEN* gene were analyzed with circBase and circInteractome. Circular RNA ID, the location of circular RNA in the genome, genomic length, spliced length and the RNAseq sources were shown

**Table 2:** Relationship between circPTEN1 and clinical characteristics in CRC patients (*n* = 150)

		Cases	Low expression	High expression	*P*
**Gender**	Male	89	46 (61%)	43 (57%)	0.5652
	Female	61	29 (39%)	32 (43%)	
**Age**	≤60	41	19 (25%)	22 (29%)	0.5241
	> 60	109	56 (75%)	53 (71%)	
**Tumor size (cm)**	≤5	28	11 (15%)	17 (23%)	0.1493
	> 5	122	64 (85%)	58 (77%)	
**Differentiation**	Well/Moderate	89	42 (56%)	47 (63%)	0.3133
	Poor	61	33 (44%)	28 (37%)	
**T stage**	T1 + T2	64	28 (37%)	36 (48%)	0.1156
	T3 + T4	86	47 (63%)	39 (52%)	
**N stage**	N0	44	15 (20%)	29 (39%)	0.0032**
	N1 + N2	106	60 (80%)	46 (61%)	
**M stage**	M0	130	61 (81%)	69 (92%)	0.0228*
	M1	20	14 (19%)	6 (8%)	
**Pathological stage**	I + II	38	12 (16%)	26 (35%)	0.0021**
	III + IV	112	63 (84%)	49 (65%)	

*p < 0.05, **p < 0.01. Using median circPTEN1 values as cutoff

### Characterization of circPTEN1 in colorectal cancer

**Fig. 2:**
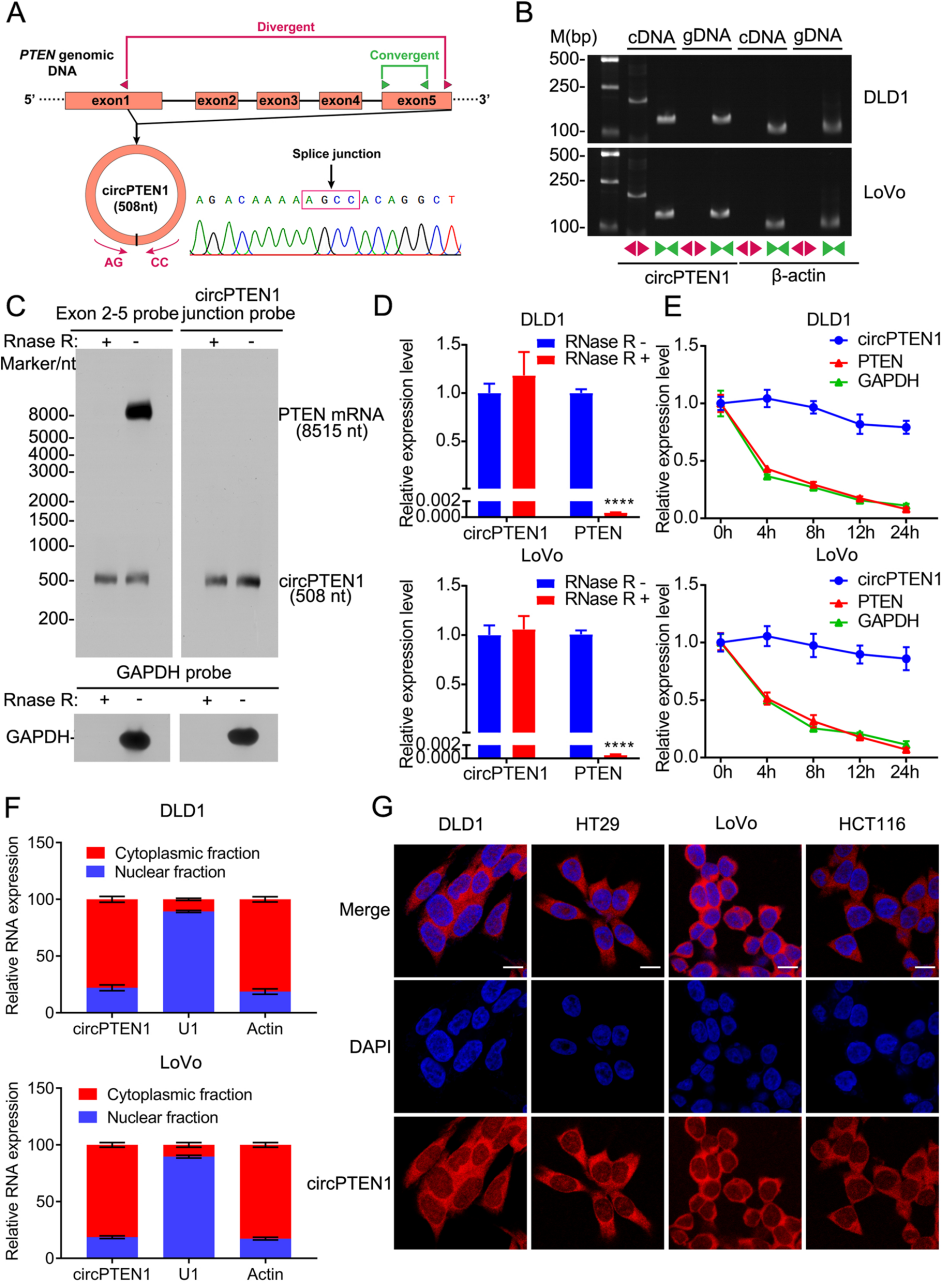
The identification and characteristics of circPTEN1 in colon cancer. **A.** Schematic illustration showing the PTEN exon 1 (partial)-exon 5 circularization forming circPTEN1. The presence of circPTEN1 was validated by RT-PCR, followed by Sanger sequencing. Black arrow represents “head-to-tail” circPTEN1 splicing sites. **B**. The presence of circPTEN1 was validated in DLD1 and LoVo cell lines by RT-PCR. Divergent primers amplified circPTEN1 in cDNA but not in genomic DNA. β-actin was used as a negative control. **C.** Northern blotting analysis of circPTEN1 and PTEN mRNA levels in LoVo cells by hybridization with exon 5 (top, left) and exon 5-exon 1 junction (top, right) probes with and without RNase R treatment. GAPDH mRNA with or without RNase R treatment was detected as a control. **D.** qRT-PCR analysis of the expression of circPTEN1 and PTEN mRNA after treatment with RNase R in DLD1 and LoVo cells. ****, P < 0.0001. **E.** qRT-PCR for the abundance of circPTEN1, PTEN mRNA and GAPDH mRNA in DLD1 and LoVo cells treated with Actinomycin D at the indicated time points. **F**. The levels of circPTEN1 in the nuclear and cytoplasmic fractions of DLD1 and LoVo cells. **G.** FISH detection of circPTEN1 in colon cancer cells. Nuclei were stained with DAPI. Scale bar, 10 μm

Owing to the distinctive expression of circPTEN1 in CRC and NAT, we postulated that it is involved in CRC progression. We thus aimed to characterize circPTEN1 by RT-PCR, Sanger sequencing, RNase R treatment, northern blot, and actinomycin D treatment in colon cancer DLD1 and LoVo cells according to previously described methodology [21–23]. circPTEN1 is generated by circularization of exons 1 to 5 of the *PTEN* gene, and its spliced mature sequence length is 508 bp according to circbase [19]. The presence of head-to-tail back-splicing junction in the circPTEN1 RT-PCR product was confirmed by Sanger sequencing (Fig. 2A). To rule out the possibility that the observed head-to-tail splicing was produced by trans-splicing, genomic rearrangements or PCR artifacts, inward-facing convergent and outward-facing divergent primers were designed to amplify PTEN mRNA and circPTEN1, respectively (Fig. 2A). The gel electrophoresis of RT-PCR products revealed that circPTEN1 was amplified by divergent primers in cDNA, but not in gDNA (Fig. 2B). Moreover, we used probes that hybridize with the splicing junction to distinguish circPTEN1 and probes that hybridize with exon 5 to distinguish circPTEN1 and its host gene, PTEN, by northern blotting (Fig. 2C), which confirmed that circPTEN1 resolved at ~ 500 nt, consistent with the hsa_circ_0002232 annotation. Resistance to digestion with RNase R exonuclease, which specifically degraded linear RNAs but not circRNAs, further validated that this RNA species harbors a cyclic structure (Fig. 2D). Treatment with actinomycin D, which can block new transcription, showed that circPTEN1 transcripts were more stable in comparison to PTEN mRNA (Fig. 2E). Further nuclear and cytoplasmic fractionation analysis demonstrated that circPTEN1 was found in both compartments, but was predominantly localized in the cytoplasm of CRC cells (Fig. 2F), which was also confirmed by the fluorescence in situ hybridization assay (FISH) (Fig. 2G and Supplementary Fig. 2). These results collectively reveal that circPTEN1 is an abundant and stable circRNA expressed in CRC that is generated from *PTEN* by back splicing.

### EIF4A3 suppresses circPTEN1 expression

**Fig. 3:**
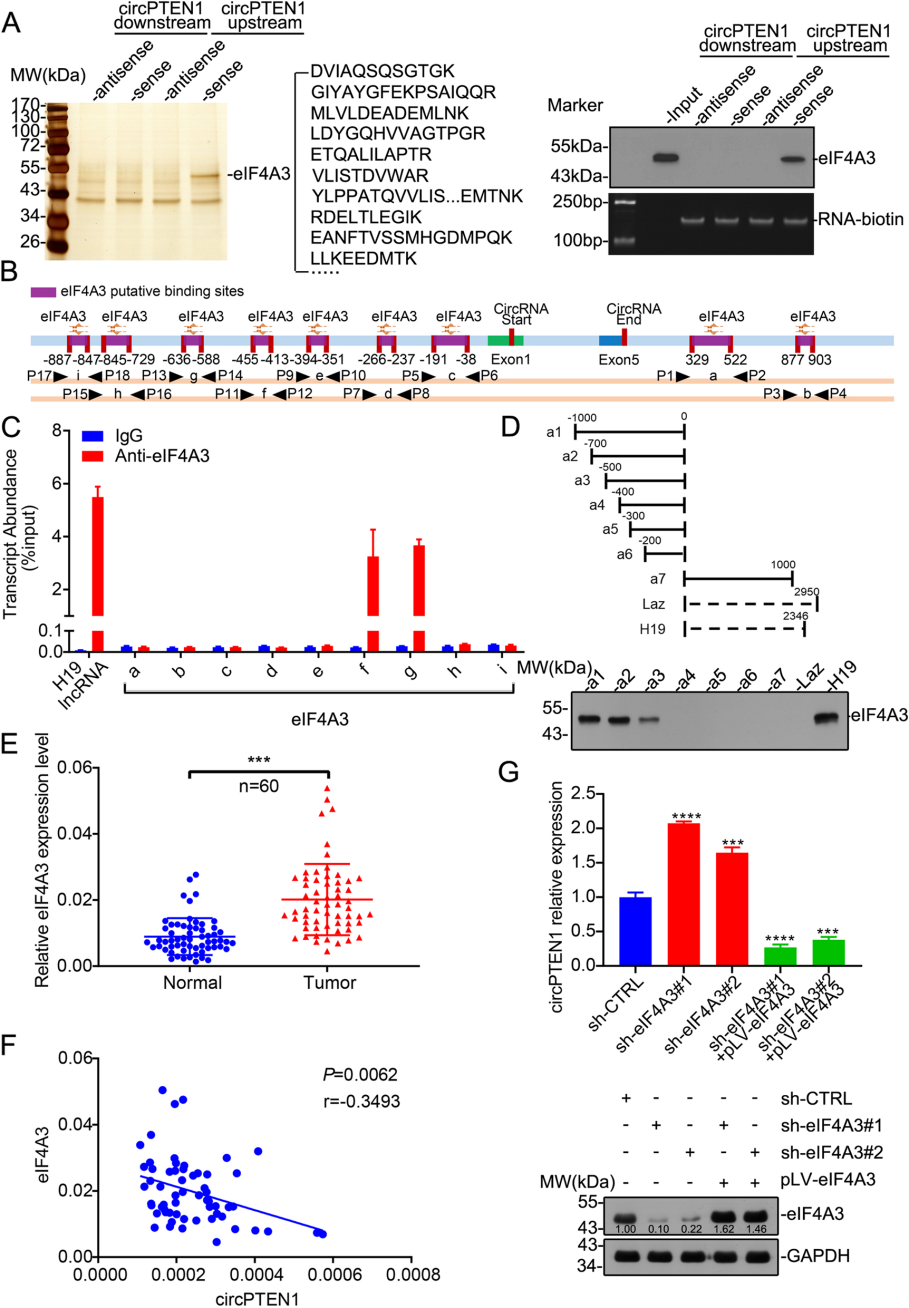
EIF4A3 suppresses circPTEN1 expression. **A**. Left, identification of proteins pulled down by circPTEN1 upstream or downstream sequences with protein extracts from LoVo cells. The arrow indicates the additional band containing eIF4A3. Right, immunoblot analysis of eIF4A3 after pull-down assay showing its specific association with circPTEN1 upstream flanking sequence (upper panel); the enriched RNA labeled with biotin was evaluated by RT-PCR (lower panel). **B.** The binding sites of eIF4A3 were predicted in the flanking region of circPTEN1 using CircInteractome. **C.** The RIP assay was performed to verify the binding sites of eIF4A3 on circPTEN1 upstream sequences. H19 lncRNA was used as the positive control. **D.** The RNA pull-down assay was performed to analyze the interaction between eIF4A3 and several circPTEN1 upstream truncations (a1-a6). Laz was a nonsense sequence used as the negative control, and H19 was used as the positive control. **E.** qRT-PCR assay showing the relative levels of eIF4A3 (normalized to β-actin) in the peritumor and tumor tissues of colon cancer (*n* = 60). ***, *P* < 0.001. **F.** The correlation between eIF4A3 and circPTEN1 (*n* = 60). **G.** LoVo cells were infected with lentivirus expressing eIF4A3 shRNA, or scramble shRNA. A rescue experiment was conducted by infecting eIF4A3-knockdown cells with pLV-eIF4A3. The expression of eIF4A3 was validated by western blot, and the ratio of the grayscale value of the eIF4A3 band to the grayscale value of the corresponding GAPDH band was labeled (lower panel). The circPTEN1 level was evaluated by qRT-PCR (upper panel)

Several RNA-binding proteins (RBPs), such as QKI, FUS and ESRP1, can bind to intronic sequences surrounding circularized exons to facilitate or suppress circularization [24]. Therefore, to explore how circPTEN1 is differentially expressed in CRC, RNA pull-down was performed to explore potential RBPs binding to flaking sequences of the circPTEN1 mRNA transcript. Mass spectrometry revealed that eIF4A3, an RBP reported to regulate exon circularization, specifically interacts with the upstream flanking sequences of circPTEN1, which was considered to be a potential circPTEN1 modulator (Fig. 3A). We further used CircInteractome to predict eIF4A3 binding sites matching the flanking regions of circPTEN1, and it revealed 7 and 2 binding sites of eIF4A3 in the upstream or downstream region of circPTEN1 mRNA, respectively (Fig. 3B). RIP assay using an anti-eIF4A3 antibody indicated that eIF4A3 can bind with circPTEN1 mRNA through two upstream putative binding sites, which we named f and g, in the corresponding RNA-protein complex (Fig. 3C and Supplementary Fig. 3), but not downstream sequences. RNA pull-down assays with RNA truncations upstream of circPTEN1 further validate the necessity of these binding sites for the interaction of circPTEN1 upstream sequences with eIF4A3 (Fig. 3D). To determine the effect of eIF4A3 on circPTEN1 cyclization, the expression of eIF4A3 in 60 paired CRC samples was examined. We found that the expression of eIF4A3 was higher in CRC tissues than in the corresponding NATs (Fig. 3E), which is consistent with the expression pattern of eIF4A3, as revealed by analyzing the publicly available databases UALCAN [25] and GEPIA [26] (Supplementary Fig. 4A and B). Furthermore, a correlation analysis was conducted, and it revealed that the level of eIF4A3 transcripts negatively correlated with circPTEN1 level, suggesting that eIF4A3 is related to circPTEN1 synthesis (Fig. 3F). We next examined circPTEN1 expression levels in eIF4A3 knockdown cells. As shown in Fig. 3G, Supplementary Fig. 5A and B, circPTEN1 expression was markedly upregulated in eIF4A3 knockdown cells, which could be rescued by ectopic reintroduction of eIF4A3, highlighting the importance of the eIF4A3 status for circPTEN1 expression. Together, these data demonstrate that circPTEN1 synthesis is closely regulated by eIF4A3 and indicate that eIF4A3 may decrease the expression of circPTEN1 by binding to flanking sequences.

### CircPTEN1 suppresses TGF-β-mediated CRC metastasis

Given the significant clinical relevance of circPTEN1 in CRC aggressiveness as revealed above, we investigated the role of circPTEN1 in regulating CRC cell metastasis. We first explored circPTEN1 expression in CRC cells and the normal human colonic epithelial cell line NCM460 and found that circPTEN1 levels were significantly lower in CRC cell lines than in NCM460 cells (Supplementary Fig. 6A), which is consistent with the results that the expression of circPTEN1 was downregulated in CRC tissues compared with the corresponding NATs. To explore the roles of circPTEN1 in CRC progression, three independent shRNAs specific for circPTEN1, which had no effect on linear PTEN mRNA, were designed and transfected into DLD1 and LoVo cells (Supplementary Fig. 6B). The knockdown efficiency of circPTEN1 was subsequently detected by qRT-PCR. As presented in Supplementary Fig. 6C, the expression of circPTEN1 was markedly reduced in CRC cells transfected with sh-circPTEN1 compared to the sh-NC group, especially in sh-circPTEN1#2-transfected cells. Thus, we chose sh-circPTEN1#2 for subsequent experiments. Additionally, a circPTEN1-overexpressing plasmid (circPTEN1-RS) was constructed in which the si-circPTEN1#2 target sequence was mutated (Supplementary Fig. 6D). CircPTEN1-RS or control plasmid was separately introduced into circPTEN1 knockdown DLD1 and LoVo cells, and the expression of circPTEN1 was sequentially detected by northern blot (Supplementary Fig. 6E). As shown in Supplementary Fig. 6F and G, the knockdown or overexpression of circPTEN1 had no effect on the expression of PTEN. We first performed Transwell assays with or without Matrigel to evaluate the effect of circPTEN1 on the migration and invasion capacity of CRC cells. However, no significant changes in cell migration and invasion capacity were observed when cells were cultured without treatment (Supplementary Fig. 7).

**Fig. 4:**
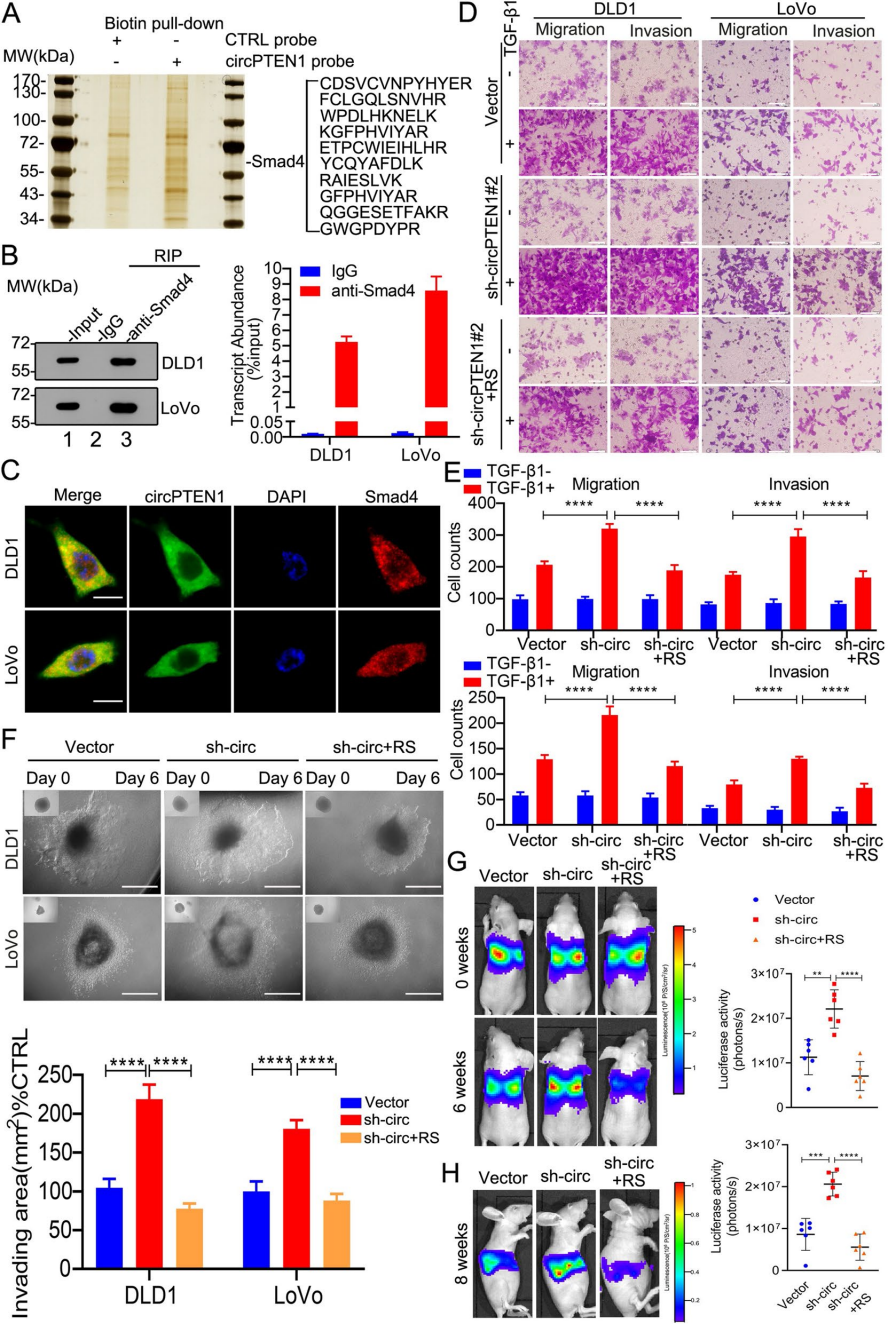
CircPTEN1 suppresses TGF-β-mediated CRC metastasis. **A.** Identification of the circPTEN1-protein complex pulled down by the circPTEN1 junction probe with protein extracts from LoVo cells. The arrow indicates the band containing Smad4. **B.** RIP assays showing the association of Smad4 with circPTEN1. Left, IP efficiency of Smad4 antibody shown by western blot. Right, relative enrichment representing RNA levels associated with Smad4 relative to an input control. IgG served as a control. **C.** Colocalization analysis of Smad4 and circPTEN1 using protein IF and RNA FISH assays, respectively. Scale bar, 10 μm. **D** and **E.** The indicated cells were treated with 5 ng/mL TGF-β1 for 48 h, and motility was sequentially evaluated through Transwell migration assays and invasion assays. (**D**) Representative images. Scale bar, 40 μm. (**E**) Migrated cells were counted in five random fields per well. Upper, DLD1. Lower, LoVo. RS: pLCDH-circPTEN1-RS. ****, *p* < 0.0001. **F.** Cells treated with TGF-β1 as indicated in Fig. 4D were subjected to the 3D multicellular tumor spheroids invasion assay. Upper, representative images. Lower, the invading area of 15 cells was analyzed per group. ****, p < 0.0001. The scale bars represent 600 μm. RS: pLCDH-circPTEN1-RS. **G** and **H.** Increased or decreased tumor metastasis formed in the lungs of mice through vein tail injection (**G**) or in the livers of mice through inferior hemispleen implantation (**H**) of circPTEN1 knockdown cells and circPTEN1 overexpression cells treated with TGF-β1 as indicated in Fig. 4D. Left panel, representative bioluminescent images. Right panel, statistical analysis of bioluminescent tracking plots. **, *p* < 0.01. ***, *p* < 0.001. ****, p < 0.0001

Considering that the activation of in vivo cancer metastasis is a complex and multistep process depending on a series of intracellular signaling networks induced by TGF-β, EGF, PDGF and other signals [27], we sought to identify interacting partners, specifically circPTEN1-associated proteins, by circRNA pull-down to investigate how circPTEN1 functions. Affinity pull-down followed by mass spectrometry revealed that Smad4, the central intracellular mediator of transforming growth factor-β/Smad (TGF-β/Smad) signaling, which is a key pathway in cancer invasion and metastasis, was one of the proteins associated with circPTEN1 with the highest abundance (Fig. 4A, Supplementary Fig. 8A and B). Moreover, bioinformatic analysis with RPISeq revealed that Smad4 was likely to interact with circPTEN1 [28]. We confirmed this interaction by performing ribonucleoprotein immunoprecipitation (RIP) analysis using an antibody that recognized Smad4 (Fig. 4B). Immunofluorescence analysis further revealed that circPTEN1 colocalized with Smad4 in the cytoplasm (Fig. 4C). The association of circPTEN1 with Smad4 indicates that circPTEN1 may affect CRC development by regulating the TGF-β/Smad signaling. We thus evaluated the effect of circPTEN1 on CRC motility with TGF-β1 treatment. The knockdown of circPTEN1 dramatically increased the migration and invasion activities induced by TGF-β1, which could be attenuated by reintroduction of circPTEN1 (Fig. 4D and E). Moreover, by using 3D morphogenesis Matrigel cultures, we observed that knockdown of circPTEN1 in CRC cells resulted in increased invasion areas, and ectopic overexpression of circPTEN1 in these cells greatly decreased the cell invasiveness under TGF-β1 treatment (Fig. 4F). We further investigated the in vivo functions of circPTEN1 in CRC cell metastasis. The results demonstrated that the bioluminescent signal detected in the lung (Fig. 4G) or liver (Fig. 4H) of mice injected with circPTEN1 knockdown cells was remarkably higher than that in the controls, and the enhanced metastatic capacity in these cells could be efficiently attenuated by reintroduction of circPTEN1. Collectively, these findings demonstrate that circPTEN1 inhibits TGF-β-mediated CRC cell aggressiveness.

### Direct interaction between circPTEN1 and Smad4 inhibits the TGF-β1/Smad signaling pathway

**Fig. 5:**
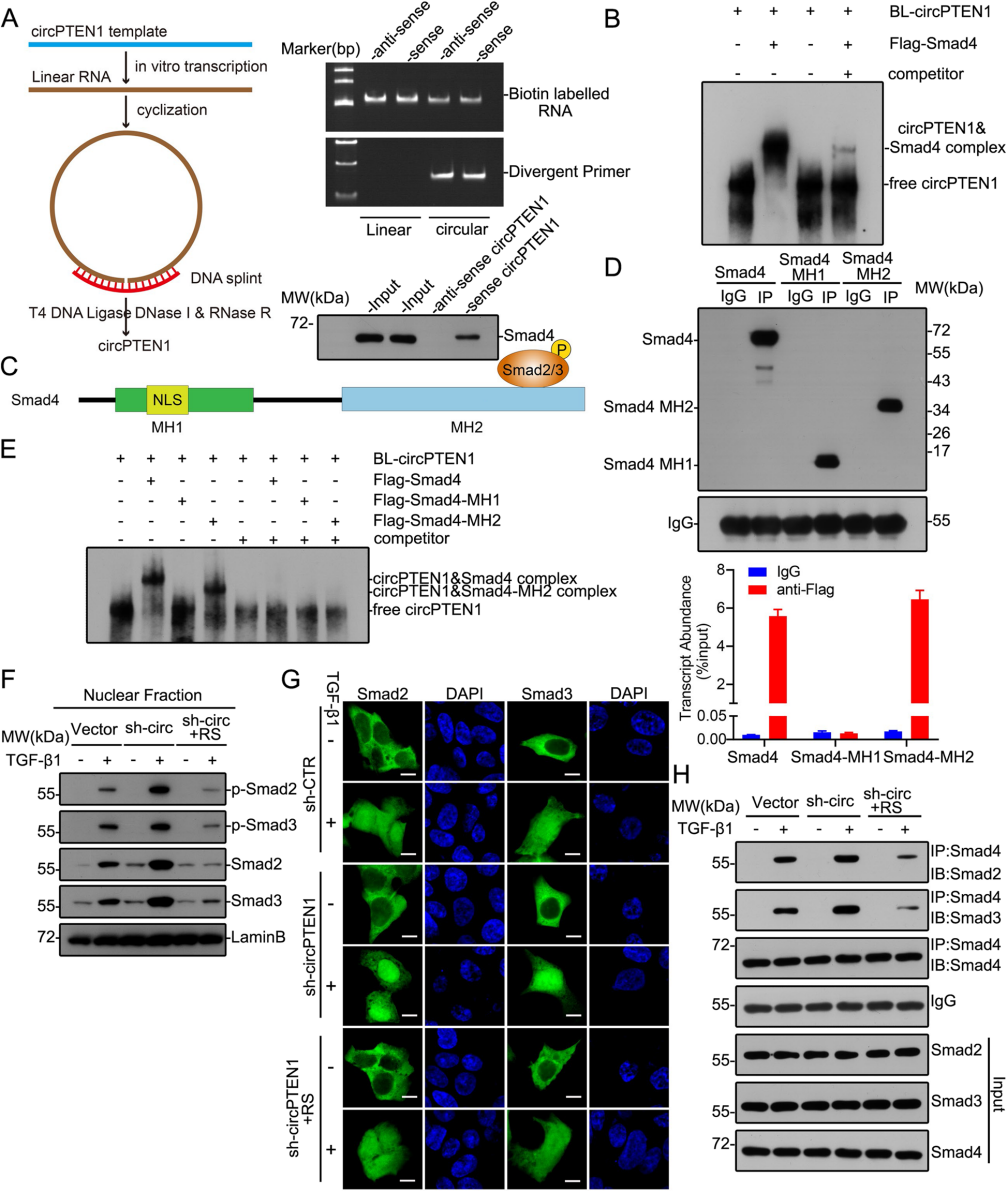
Direct interaction between circPTEN1 and Smad4 inhibits the TGF-β1/Smad signaling pathway. **A.** Left, schematic illustrating the cyclization of linear RNA generated in vitro. Right, top, RT-PCR analysis of linear and cyclized circPTEN1 RNAs. Right, bottom, RNA-protein pull-down assays were conducted with FLAG-Smad4 purified from 293 T cells transfected with pCMV-tag-2b-Smad4 against cyclized circPTEN1. **B.** Electrophoretic mobility shift analysis of interactions between circPTEN1 and FLAG-Smad4. **C.** The Smad4 protein is characterized by the presence of Mad homology domain-1 (MH1) and Mad homology domain-2 (MH2). **D.** RIP assays showing the association of full-length Smad4 or Smad4 truncations with circPTEN1. Top, IP efficiency of FLAG-antibody shown by western blot. Down, relative enrichment representing RNA levels associated with full-length Smad4 or Smad4 truncations relative to an input control. IgG antibody served as a control. **E.** Electrophoretic mobility shift analysis of interactions between circPTEN1 and FLAG-Smad4 or FLAG-Smad4 truncations. **F.** The distribution of Smad2, Smad3, p-Smad2 and p-Smad3 in the nuclear fraction. LoVo cells with circPTEN1 knockdown or overexpression as indicated in Supplementary Fig. 6C and 6E were treated with 5 ng/mL TGF-β1 for 1 h. Nuclear proteins were extracted to detect the expression of the indicated proteins. Lamin B was used as a nuclear marker. RS: pLCDH-circPTEN1-RS. **G.** Subcellular localization of Smad2 or Smad3. C-terminal GFP-tagged Smad2 or Smad3 was introduced into circPTEN1 knockdown or overexpression LoVo cells, prior to be treated with 5 ng/mL TGF-β1 for 1 h. Cells were then stained with DAPI, followed by imaging with confocal microscopy. The scale bars represent 5 μm. RS: pLCDH-circPTEN1-RS. **H.** circPTEN1 inhibits the formation of Smad2-Smad4 and Smad3-Smad4 complexes. Western blot analysis of Smad2 and Smad3 following immunoprecipitation of Smad4 from circPTEN1 knockdown or overexpression LoVo cells as indicated in Supplementary Fig. 6C and 6E, which were treated with 5 ng/mL TGF-β1 for 1 h. RS: pLCDH-circPTEN1-RS

We sought to explore the mechanism by which circPTEN1 is involved in TGF-β1-mediated CRC progression. The results of circRNA pull-down and RIP mentioned above indicate that circPTEN1 may interact with Smad4. Reconstructing these interactions in vitro demonstrated that purified Smad4 was able to coprecipitate with cyclized biotin-labeled circPTEN1 (Fig. 5A). Moreover, the interaction of Smad4 with circPTEN1 was readily detected by supershift in electrophoretic mobility shift assays (Fig. 5B). These results confirmed that circPTEN1 directly interacts with Smad4. The Smad4 protein is characterized by two conserved regions known as N-terminal Mad homology domain-1 (MH1) and C-terminal Mad homology domain-2 (MH2) [29]. The MH1 domain contains a nuclear localization signal (NLS), and the MH2 domain binds to several transcription factors, including Smad2/3 (Fig. 5C). RIP assays using Smad4 truncation mutants indicated that circPTEN1 directly binds to Smad4 via the MH2 domain (Fig. 5D). This interaction of circPTEN1 with the MH2 domain of Smad4 was further confirmed using EMSA (Fig. 5E). To further investigate which specific region within circPTEN1 contributes to Smad4 binding, we constructed five different deletion mutants of circPTEN1 (Supplementary Fig. 9A). Subsequently, RNA pull-down assay followed by western blot showed that the 451–50 nt deletion mutant of circPTEN1 could not interact with Smad4, while the capacity of the other circPTEN1 truncations to bind Smad4 was basically consistent with the full-length circPTEN1 (Supplementary Fig. 9B), indicating that the 451–50 nt-long region that covers the head-to-tail back-splicing junction renders circPTEN1 able to bind to Smad4. Moreover, we found that the dramatically increased migration and invasion activities of circPTEN1 knockdown LoVo cells induced by TGF-β1 could be attenuated by reintroduction of full-length circPTEN1 but not the 451–50 nt deletion mutant of circPTEN1 (Supplementary Fig. 9C and D), demonstrating that the effect of circPTEN1 on the migration and invasion activities of CRC mediated by TGF-β1 was dependent on its interaction with Smad4.

TGF-β interacts with TGF-β receptors to activate R-Smads (Smad2 and Smad3), which in turn form a heteromeric complex with Smad4. These complexes are subsequently translocated to the nucleus to regulate the expression of target genes. The results, as mentioned above, showed that circPTEN1 interacts with Smad4 through its MH2 domain, which also serves as the binding site for R-Smad/Smad4 interactions. Therefore, we hypothesize that circPTEN1 could modify the TGF-β-induced nuclear translocation of Smad2 and Smad3. As shown in Fig. 5F and Supplementary Fig. 10, the knockdown of circPTEN1 resulted in increased levels of nuclear Smad2 and Smad3 relative to that of control cells in response to TGF-β1, and the opposite response was observed in circPTEN1-reintroduced cells relative to that of circPTEN1 knockdown cells. Moreover, this negative correlation between circPTEN1 levels and nuclear Smad2/3 was further demonstrated by immunofluorescence analysis (Fig. 5G). Sequentially, the role of circPTEN1 in blocking the interactions of Smad4 with either Smad2 or Smad3 was examined to determine the mechanism by which circPTEN1 suppresses the nuclear retention of Smad2 and Smad3. We observed that the knockdown of circPTEN1 led to a dramatic increase in Smad2/Smad3 and Smad3/Smad4 complexes mediated by TGF-β1, which could be attenuated by reintroduction of circPTEN1 (Fig. 5H). These results demonstrate that circPTEN1 inhibits the formation of R-Smad/Smad4 complexes, thereby suppressing their translocation and ultimately downregulating the expression of TGF-β1 target genes.

### circPTEN1 suppresses CRC metastasis by inhibiting TGF-β/Smad-mediated EMT

Transforming growth factor-β (TGF-β) plays a dual role as a potent growth inhibitor of epithelial and nonepithelial cells, inducing growth arrest and apoptosis, and as a promoter of tumor progression in advanced cancers, inducing epithelial-mesenchymal transition (EMT).

**Fig. 6:**
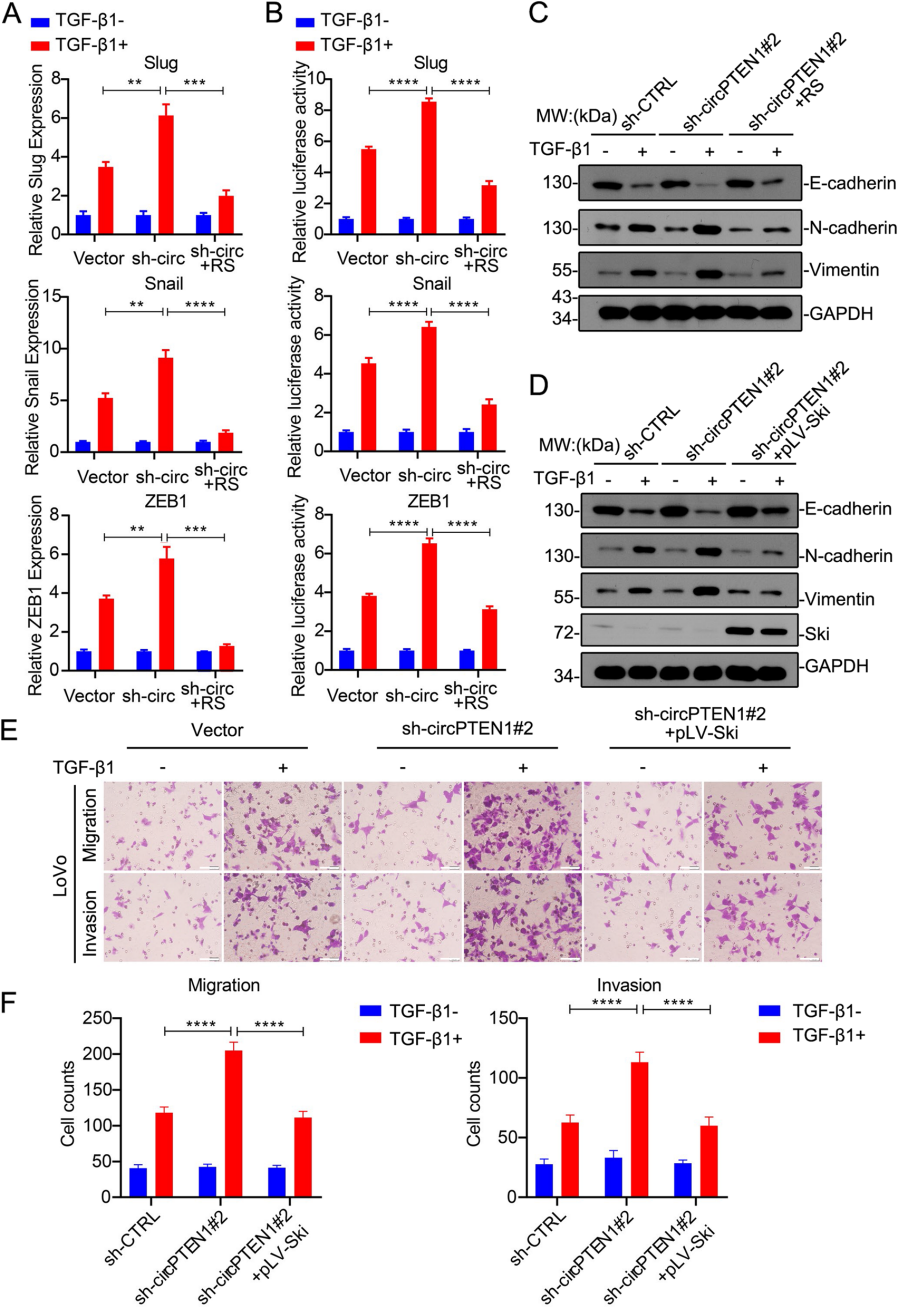
circPTEN1 suppresses CRC metastasis by inhibiting TGF-β/Smad-mediated EMT. **A.** The expression of Snail, Slug and ZEB1 in circPTEN1 knockdown or overexpressed LoVo cells as indicated in Supplementary Fig. 6C and Supplementary Fig. 6E treated with TGF-β1 was analyzed by qRT-PCR. RS: pLCDH-circPTEN1-RS. **, *p* < 0.01. ***, *p* < 0.001. ****, *p* < 0.0001. **B.** Dual-luciferase reporter assays of LoVo cells as indicated in Supplementary Fig. 6C and Supplementary Fig. 6E transfected with Snail, Slug or ZEB1 promoters in the presence or absence of TGF-β1 for 24 h. RS: pLCDH-circPTEN1-RS. **C.** LoVo cells, as indicated in Supplementary Fig. 6C and Supplementary Fig. 6E were treated with TGF-β1 for 24 h. The expression of E-cadherin, N-cadherin and vimentin was determined by immunoblot analysis. RS: pLCDH-circPTEN1-RS. **D.** circPTEN1 knockdown LoVo cells, circPTEN1 knockdown LoVo cells overexpressing Ski, or control cells were treated with TGF-β1 for 24 h. The expression of E-cadherin, N-cadherin, vimentin and Ski was determined by immunoblot analysis. **E.** circPTEN1 knockdown LoVo cells, circPTEN1 knockdown LoVo cells overexpressing Ski, or control cells were treated with TGF-β1 for 48 h, and the motility of these cells was sequentially evaluated through Transwell migration assays and invasion assays. Scale bar, 40 μm. **F.** Migrated cells in Fig. 6E were counted in five random fields per well to calculate cell migration and invasion ability. ****, *p* < 0.0001

The migratory and invasive properties imparted by EMT are known to be essential for metastasis. The results circPTEN1 inhibits the formation of R-Smad/Smad4 complexes and the nuclear translocation of Smad2/3 as mentioned above indicate that circPTEN1 may suppress CRC metastasis by inhibiting TGF-β1/Smad-mediated EMT. We first detected the effect of circPTEN1 on the expression of EMT-related genes induced by TGF-β1. CircPTEN1 knockdown enhanced the expression of TGF-β1/Smad signaling-targeted EMT genes compared with control cells, which could be attenuated by reintroduction of circPTEN1 (Fig. 6A). Furthermore, we found that knockdown of circPTEN1 dramatically enhanced TGF-β1-induced transcriptional activity of Snail, Slug or ZEB1-luciferase reporter, and these elevated transcriptional activation could be compromised by reintroduction of circPTEN1 (Fig. 6B). We then detected the protein levels of epithelial markers and mesenchymal markers. With TGF-β1 treatment, the loss of epithelial markers and gain of mesenchymal markers were enhanced by circPTEN1 knockdown, and the opposite response was detected with circPTEN1 overexpression (Fig. 6C). These results indicate that circPTEN1 suppresses the EMT induced by TGF-β1. Ski is a well-known suppressor of TGF-β signaling, and it disrupts the formation of a functional heteromeric Smad complex in response to TGF-β between the Co- and R-Smads through binding to either component [30]. We found that the elevated transcription of Snail, Slug or ZEB1 and the enhanced motility capacity of CRC cells resulting from circPTEN1 knockdown could be drastically inhibited by Ski overexpression (Fig. 6D, E and F), further confirming that circPTEN1 suppresses CRC metastasis by inhibiting TGF-β1/Smad-mediated EMT.

Our results demonstrate that circPTEN1 inhibits TGF-β1/Smad-mediated EMT by downregulating the expression of EMT-related genes, including Snail. The PI3K/AKT axis was reported to upregulate the expression of Snail by regulating different molecular pathways, such as the PI3K/AKT/mTOR/NF-kB pathway [31]. Considering that the best characterized function of the PTEN protein is the ability to dephosphorylate PtdIns (3,4,5) P3 and convert it back into PIP2 in the cytoplasm [32], it may attenuate the upregulation of Snail resulting from circPTEN1 knockdown by suppressing the PI3K/AKT pathway. We thus evaluated the effect of PTEN levels on circPTEN1-mediated suppression of cancer progression. As shown in Supplementary Fig. 11B and C, under TGF-β1 treatment, the increased migration and invasion activities of LoVo cells resulting from circPTEN1 knockdown could be attenuated by PTEN overexpression. Moreover, the suppressive effect of circPTEN1 overexpression on cancer cell invasiveness could be efficiently relieved by knockdown of PTEN (Supplementary Fig. 12B and C). We further measured the expression of Snail in the aforementioned LoVo cells. As shown in Supplementary Fig. 11D, knockdown of circPTEN1 dramatically enhanced TGF-β1-induced transcriptional activity of Snail, and this elevated transcriptional activation could be compromised by PTEN overexpression. Furthermore, knockdown of PTEN relieved the suppressive effect of circPTEN1 overexpression on the transcription of Snail (Supplementary Fig. 12D). These results indicate that the effects of PTEN levels on the invasiveness of circPTEN1 overexpressing or knockdown LoVo cells may be partially attributed to regulating the expression of Snail, and demonstrate that different family members of the *PTEN* gene, circPTEN1 and the PTEN protein, could both inhibit CRC metastasis, which highlights the complexity of the mechanism by which the *PTEN* gene exerts tumor-suppressor functions.

## Discussion

As one of the most frequently mutated tumor suppressor genes in human cancer, *PTEN* aberrations, such as mutations and copy number variation, are widely involved in the progression of multiple human cancers [33]. The most well-studied tumor suppression role of canonical PTEN protein is to counteract the PI3K/Akt signaling cascade by dephosphorylating PtdIns (3,4,5) P3 and converting it back into PIP2 in the cytoplasm to control cell proliferation/invasiveness and promote apoptosis [32]. In addition to its tumor suppressor function, *PTEN* is also involved in various biological processes, including cell metabolism and antiviral innate immunity [1]. In addition to the canonical PTEN, three recently identified N-terminally extended PTEN isoforms were demonstrated to exert distinct biological functions, and they are reported to play multiple roles in tumorigenesis [3–5]. However, the results of PTEN isoforms in cancer development from these studies are mutually contradictory [6, 8]. Nonetheless, the current exploration of the canonical PTEN and PTEN isoforms cannot fully illustrate the diversity of their involvement in biological processes and tumor development. In the current study, we explored the potential of *PTEN* gene-generating circRNAs to exert functions. A circRNA derived from the *PTEN* gene (circPTEN1) was identified as a suppressor of CRC progression. We further verified that circPTEN1 is significantly downregulated in CRC and suppresses CRC metastasis mediated by TGF-β/Smad signaling by interacting with Smad4.

Circular RNAs (circRNAs) are a class of single-stranded closed RNA molecules that are formed by precursor mRNA back-splicing. Next-generation RNA sequencing and bioinformatics analysis have discovered that circRNAs are widely expressed across the eukaryotic tree of life, and it has been reported that nearly 10% of genes transcribed in eukaryotic cells can produce circRNAs to exert functions [34]. As one of the most frequently mutated genes in human cancer, previous studies of *PTEN* mainly focus on exploration of the tumor suppression role of the canonical PTEN protein and the N-terminal extended PTEN isoforms, while fewer studies on noncoding RNAs arising from *PTEN* have been reported. We sought to uncover circRNAs arising from the *PTEN* gene and explore their involvement in tumorigenesis. We demonstrate that circPTEN1 is a circular RNA generated from the *PTEN* gene by back-splicing, which suppresses CRC metastasis.

CircRNA production is highly dependent on biological settings and tightly regulated in cells using different *cis* elements and *trans* factors that are specific for back-splicing [35]. Several RBPs have been reported to facilitate or suppress circRNA biogenesis. For instance, QKI was found to enhance circRNA formation during EMT by binding to its consensus target single-stranded RNA motif in introns flanking circRNA-forming exons [36]. Moreover, the enzyme adenosine deaminase 1 acting on RNA (ADAR1) was reported to suppress circRNA expression by A-to-I editing of RNA pairs flanking circularized exon(s), which diminishes the complementarity and stability of these RNA pairs [37]. However, the functions and underlying mechanisms of most RBPs in regulating circRNA generation remain elusive. In this study, we found that the expression of eIF4A3 was higher in CRC tissues than in the corresponding NATs, and a correlation analysis revealed that the level of eIF4A3 transcripts was negatively correlated with the circPTEN1 level. We further demonstrated that eIF4A3 could bind to the upstream flanking region of the circPTEN1 transcript and suppress its expression. Notably, eIF4A3 was previously reported to induce the expression of circASAP1 and circMMP9 in glioblastoma [38, 39]. These studies together with our results revealed that eIF4A3 was the first RBP identified to function as both suppressive and promoting roles in circRNA genesis dependent on cancer types, although the underlying mechanism is still unknown. Additionally, the multiple roles of a single RBP in circRNA genesis may partially account for the distinct profiling of circRNA expression in different tissues.

CircRNAs perform a wide variety of biological functions in eukaryotic cells by acting as sponging miRNAs, interacting with RNA binding proteins, modulating the stability of mRNAs, regulating gene transcription and translating proteins. Recent studies have shown that circRNAs play a crucial role in the carcinogenesis, progression, and metastasis of tumors [40]. Colon cancer is a malignant cancer with an increasingly high prevalence and is the fourth leading cause of cancer-related death worldwide. Approximately 20% of patients show metastasis at the time of diagnosis, with the liver being one of the most affected organs. Although comprehensive therapies have been used, the prognosis of colon cancer is still poor, which may be due to the lack of early diagnosis and effective targeted therapy agents [41]. Therefore, efficient diagnosis and therapeutic approaches are important for colon cancer research. Recently, circRNAs have been reported to widely participate in CRC pathogenesis. Zhou et al. found that circRNA_100859 functions as an oncogene in colon cancer by sponging the miR-217-HIF-1α pathway [41]. Zheng et al. showed that a protein encoded by the circular RNA circPPP1R12A promotes tumor pathogenesis and metastasis of colon cancer by modulating the Hippo-YAP signaling [42]. Moreover, circPTK2 was identified as a novel therapeutic target for metastatic colorectal cancer by Yang et al. [43]. However, the role of most circRNAs in the progression and pathogenesis of CRC is largely unknown. We found that circPTEN1 is significantly downregulated in colon cancer tissues and cell lines. Notably, patients with lower circPTEN1 expression showed a greater tendency toward lymph node metastases or distant metastases and had a significantly shorter overall survival than patients with higher circPTEN1 expression. In vitro and in vivo experiments showed that circPTEN1 inhibited the migration and invasion of CRC cells. Thus, our results revealed that circPTEN1 may be a potential therapeutic target and prognostic biomarker for CRC metastasis prevention.

Despite a primary tumor suppressor role, there is compelling evidence demonstrating that TGF-β promotes invasion and metastasis in advanced stages of colorectal cancer [43]. Alterations in TGF-β pathway components are first detected in advanced adenomas and affect 40–50% of all CRCs [44]. TGF-β has been recognized as a major EMT inducer, and it has been extensively explored for its functions as a major propeller of TGF-β-induced EMT in cancer progression and metastasis, including activation of signaling pathways and transcriptional regulators for both Smad and non-Smad pathways [45]. TGF-β interacts and ultimately phosphorylates its membrane-bound receptor, which in turn phosphorylates Smad2/3. Subsequently, p-R-Smad2/3 binds to Co-Smad4, and the activated Smad complex translocates to the nucleus and binds directly to Smad-binding DNA elements (SBEs) to turn on and off the transcription of responsive genes [45]. Increasing studies have demonstrated that TGF-β accelerates the metastasis of colon cancer by upregulating the expression of EMT-promoting genes, such as Snail and ZEB1 [46]. The rich evidence accumulated on the various mechanisms that mediate EMT after TGF-β stimulation has logically led to many attempts to identify chemical modifiers of this process, such as TGF-β receptor kinase inhibitors or anti-TGF-β ligand traps, that could be developed into clinically useful anti-metastatic drugs [47]. However, the role of circRNAs in the TGF-β/Smad signaling pathway remains largely unknown. Here, we showed that circPTEN1 binds the MH2 domain of Smad4 to disrupt its physical interaction with Smad2/3, which restricts the formation and nucleus translocation of Smad complexes, and consequently suppresses the transcription of EMT genes under TGF-β1 stimulation. Thus, circPTEN1 may be a novel strategy that can be used to suppress TGF-β/Smad signaling-mediated CRC metastasis.

Activation of the PI3K/AKT axis is emerging as a central feature of EMT. It could upregulate the expression of Snail by regulating different molecular pathways, such as the PI3K/AKT/mTOR/NF-kB pathway [31] and the PI3K/AKT/GSK-3β signaling pathway [48]. Thus, PI3K/AKT functions as a molecular switch for many signaling pathways that lead to EMT. Notably, the best characterized function of the canonical PTEN protein is to antagonize the PI3K/AKT pathway, which is mainly involved in the regulation of cell growth and invasion [2]. Thus, PTEN may regulate the expression of Snail by suppressing the PI3K/AKT pathway. In the current study, we demonstrate that PTEN overexpression suppressed the elevated metastasis capacity resulting from circPTEN1 knockdown, and the suppressive effect of circPTEN1 overexpression on cancer cell invasiveness could be efficiently relieved by knockdown of PTEN. We further showed that the effects of PTEN levels on the invasiveness of circPTEN1 knockdown or overexpressing LoVo cells may be partially attributed to regulation of Snail expression. Collectively, our results indicate that circPTEN1 and the PTEN protein are both involved in the suppression of CRC metastasis, while the differences in the mechanisms by which these two molecules regulate the expression of EMT-related genes remain to be further investigated.

In the current study, we demonstrate that circPTEN1 suppresses TGF-β/Smad signaling-mediated CRC metastasis. Notably, the TGF-β/Smad signaling pathway was reported to be associated with the metastasis of various human cancers, including breast cancer and pancreatic cancer besides colon cancer [49]. Thus, circPTEN1 may also modulate the metastasis of these cancers. Beyond tumor metastasis, the TGF-β/Smad signaling is widely involved in a variety of biological functions, including cell proliferation, differentiation, cell survival, angiogenesis and immune surveillance [43]. Our results that circPTEN1 disrupts the formation of the Smad complex induced by TGF-β1 indicate that it may be involved in these biological processes, which remains for future investigation.

## Conclusions

In conclusion, our study demonstrated that a circRNA derived from the *PTEN* gene (circPTEN1) suppresses CRC progression. We also identified Smad4 as the target of circPTEN1 and showed that circPTEN1 inhibits TGF-β-mediated CRC metastasis by disrupting TGF-β/Smad signaling through binding the MH2 domain of Smad4 to disrupt its physical interaction with p-Smad2/3. Increasing studies have shown that therapeutic strategies aimed at blocking TGF-β signaling can impair the process of CRC metastasis [50, 51]. Thus, circPTEN1 is likely to be a potential therapeutic target for CRC metastasis prevention by targeting the TGF-β signaling. It has been found that one gene locus can produce multiple circRNAs, with mechanisms related to alternative back-splicing and alternative splicing site election [52], raising the possibility that the *PTEN* gene may generate additional as yet unidentified circRNAs. Identification of circRNAs arising from the *PTEN* gene advances our understanding of the diversity and complexity of *PTEN* functions in physiological and pathological processes.

## Supplementary Information

**Additional file 1: Supplementary Fig. 1.** The expression of circPTEN2 in CRC tissues and the corresponding NATs was evaluated by FISH. **Supplementary Fig. 2.** A negative control probe was adopted and the FISH experiments were performed in CRC cells. **Supplementary Fig. 3.** The RIP assay was performed to verify the binding sites of eIF4A3 on circPTEN1 upstream sequences. **Supplementary Fig. 4.** The expression of eIF4A3 was higher in CRC tissues compared with the corresponding NATs. **Supplementary Fig. 5.** circPTEN1 synthesis is closely regulated by the eIF4A3. **Supplementary Fig. 6.** The establishment of circPTEN1 knockdown or overexpressing colon cancer cell lines. **Supplementary Fig. 7.** The effect of circPTEN1 level on cell motility without treatment. **Supplementary Fig. 8.** Smad4 was the protein associated with circPTEN1 with the highest abundance. **Supplementary Fig. 9.** The effect of circPTEN1 on the migration and invasion activities of CRC mediated by TGF-β was dependent on its interaction with Smad4. **Supplementary Fig. 10.** The expression of p-Smad2 and p-Smad3 in whole cell lysate of indicated cells. **Supplementary Fig. 11.** The effect of overexpressed PTEN on the invasiveness of circPTEN1 knockdown LoVo cells. **Supplementary Fig. 12.** The effect of PTEN knockdown on the invasiveness of LoVo cells overexpressing circPTEN1. **Supplementary Table 1.** A list of antibodies used in this study. **Supplementary Table 2.** Oligonucleotides sequences used in this study.

## Data Availability

The datasets used and/or analyzed during the current study are available from the corresponding author on reasonable request.

## Abbreviations

CRC: Colorectal cancer
circRNA: Circular RNA
EMT: Epithelial-to-mesenchymal transition
FISH: Fluorescence in situ hybridization
qRT-PCR: Quantitative real-time PCR
RIP: RNA immunoprecipitation
RBP: RNA-binding protein
EIF4A3: Eukaryotic translation initiation factor 4A3
