# Supplementary material for: circPTEN1, a circular RNA generated from PTEN, suppresses cancer progression through inhibition of TGF-β/Smad signaling
Source: Mol Cancer. 2022 Feb 8;21:41. doi: 10.1186/s12943-022-01495-y (PMC8822707; doi:10.1186/s12943-022-01495-y)
Supplement: Supplementary file 1 — Additional file 1: Supplementary Fig. 1. The expression of circPTEN2 in CRC tissues and the corresponding NATs was evaluated by FISH. Supplementary Fig. 2. A negative control probe was adopted and the FISH experiments were performed in CRC cells. Supplementary Fig. 3. The RIP assay was performed to verify the binding sites of eIF4A3 on circPTEN1 upstream sequences. Supplementary Fig. 4. The expression of eIF4A3 was higher in CRC tissues compared with the corresponding NATs. Supplementary Fig. 5. circPTEN1 synthesis is closely regulated by the eIF4A3. Supplementary Fig. 6. The establishment of circPTEN1 knockdown or overexpressing colon cancer cell lines. Supplementary Fig. 7. The effect of circPTEN1 level on cell motility without treatment. Supplementary Fig. 8. Smad4 was the protein associated with circPTEN1 with the highest abundance. Supplementary Fig. 9. The effect of circPTEN1 on the migration and invasion activities of CRC mediated by TGF-β was dependent on its interaction with Smad4. Supplementary Fig. 10. The expression of p-Smad2 and p-Smad3 in whole cell lysate of indicated cells. Supplementary Fig. 11. The effect of overexpressed PTEN on the invasiveness of circPTEN1 knockdown LoVo cells. Supplementary Fig. 12. The effect of PTEN knockdown on the invasiveness of LoVo cells overexpressing circPTEN1. Supplementary Table 1. A list of antibodies used in this study. Supplementary Table 2. Oligonucleotides sequences used in this study. [file 12943_2022_1495_MOESM1_ESM.pdf]

Supplementary materials

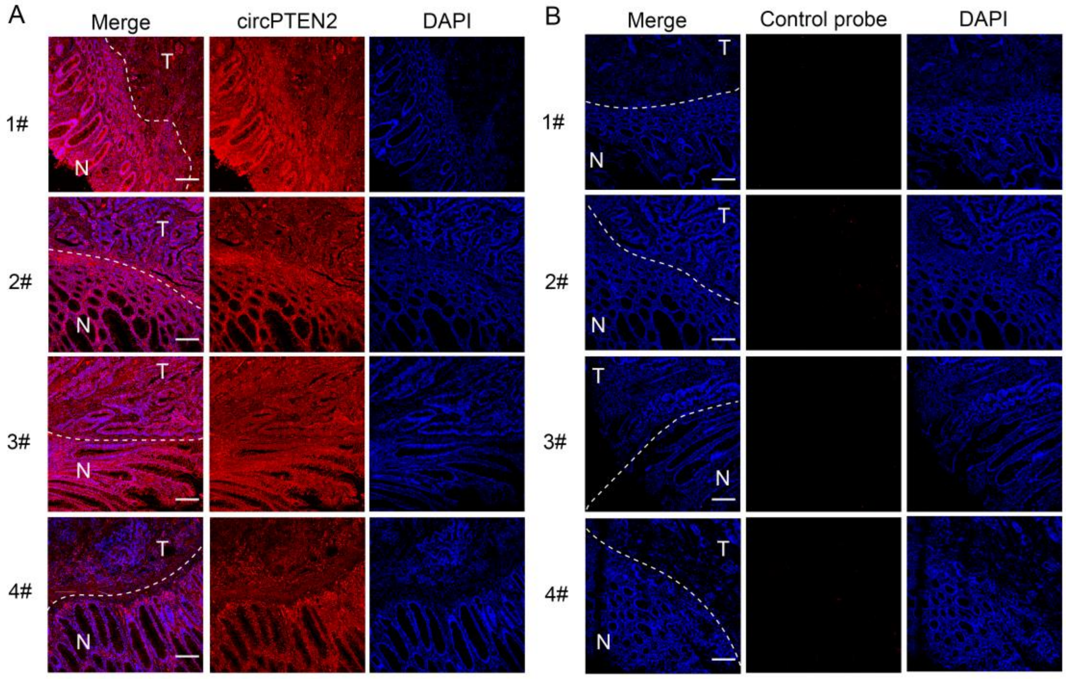

Supplementary figure 1. The expression of circPTEN2 in CRC tissues and the corresponding NATs was evaluated by FISH.

A. Fluorescence in situ hybridization assay (FISH) was conducted to determine the expression of circPTEN2 in the peritumor and tumor tissues of colon cancer. N: peri-tumor tissue, T: tumor tissue. The scale bars represent 200 μm.

B. A negative control probe was used to perform FISH in CRC tissues. N: peri-tumor tissue, T: tumor tissue. The scale bars represent 200 μm.

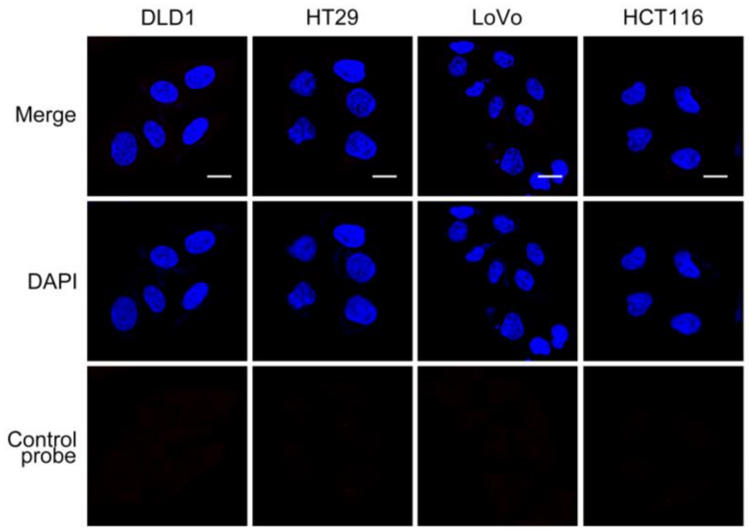

Supplementary figure 2. A negative control probe was adopted, and FISH experiments were performed in CRC cells.

Fluorescence in situ hybridization assays were conducted with a negative control probe in CRC cells. Scale bar, 10 μm.

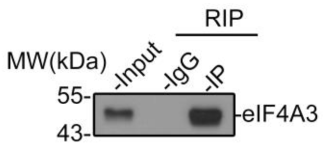

Supplementary Figure 3. The RIP assay was performed to verify the binding sites of eIF4A3 on circPTEN1 upstream sequences.

The IP efficiency of the eIF4A3 antibody was shown by western blot. IgG antibody served as a control.

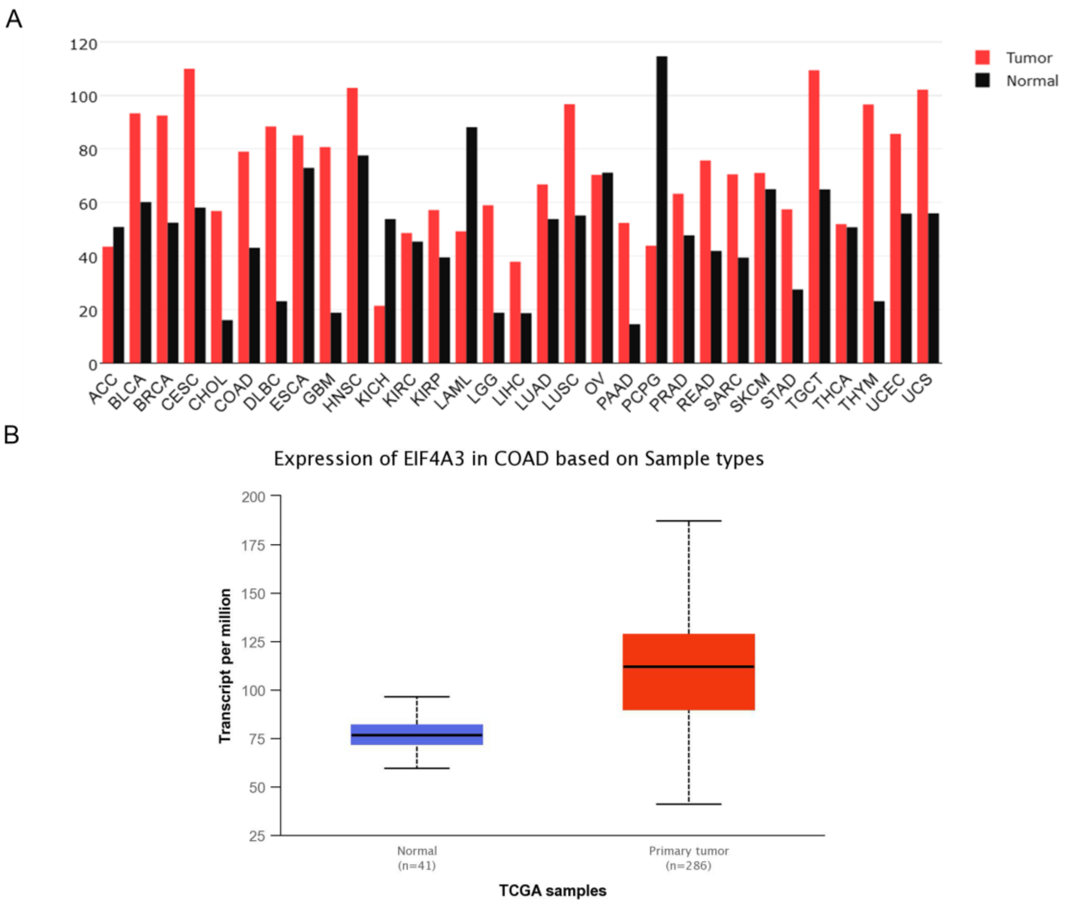

Supplementary Figure 4. The expression of eIF4A3 was higher in CRC tissues than in the corresponding NATs.

The expression pattern of eIF4A3 was revealed by analyzing the publicly available databases UALCAN(*1*) (http://ualcan.path.uab.edu/index.html) (Supplementary Figure 4A) and GEPIA(*2*) (http:// http://gepia.cancer-pku.cn/) (Supplementary Figure 4B).

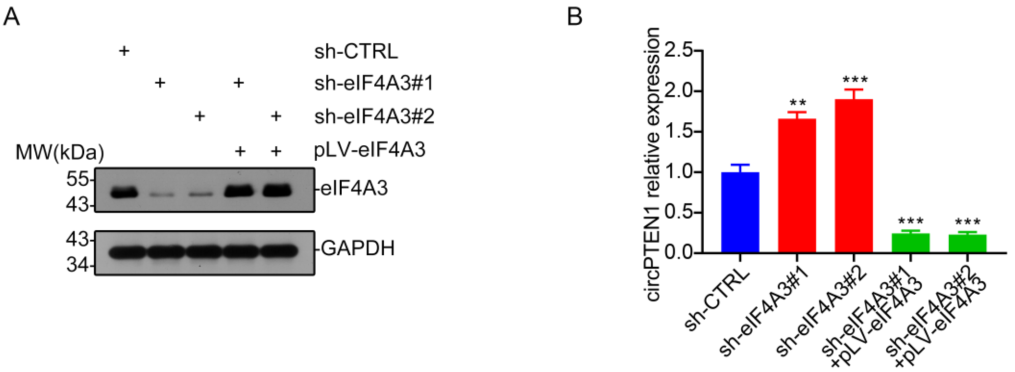

Supplementary Figure 5. circPTEN1 synthesis is closely regulated by eIF4A3.

A. To knockdown eIF4A3, DLD1 cells were infected with lentivirus expressing eIF4A3 shRNA or scramble shRNA separately. DLD1 cells with eIF4A3 knockdown were sequentially infected with lentivirus expressing FLAG-eIF4A3. The knockdown or rescue of eIF4A3 was validated by western blot. GAPDH was used as a loading control.

B. The expression of circPTEN1 in eIF4A3 knockdown or overexpressing DLD1 cells as indicated in S5A was evaluated by qRT-PCR. **, $p<0.01$. ***, $p<0.001$.

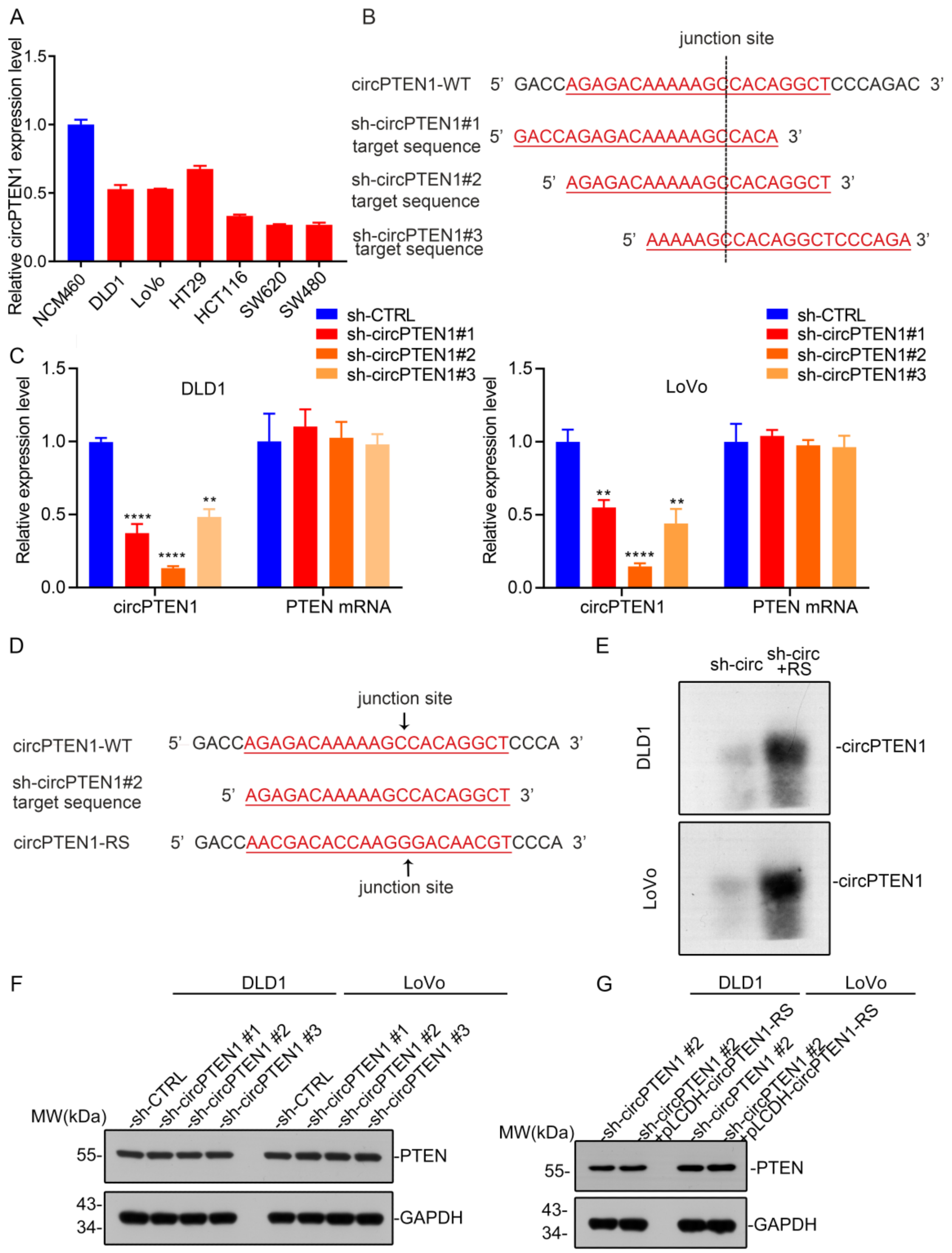

Supplementary Figure 6. The establishment of circPTEN1 knockdown or overexpressing colon cancer cell lines.

A. The expression of circPTEN1 in the normal human colonic epithelial cell line NCM460 and a panel of human colon cancer cell lines was evaluated by qRT-PCR.

B. The target sequences used to knockdown circPTEN1 are shown. The junction site of circPTEN1 is labeled with a black-dotted line.

C. DLD1 (left) and LoVo (right) cells were infected with lentivirus expressing circPTEN1 shRNA, or scramble shRNA separately. The knockdown of circPTEN1 was validated by qRT–PCR. **, p<0.01. ****, p<0.0001.

D. The sequences of circPTEN1-RS flanking the junction site are shown.

E. The circPTEN1-RS transcript was inserted into pLCDH-ciR, and the expression of circPTEN1-RS in pLCDH-circPTEN1-RS-overexpressing cells was evaluated with northern blotting. sh-circ: sh-circPTEN1#2; RS: pLCDH-circPTEN1-RS.

F and **G.** The expression of PTEN in circPTEN1 knockdown (**F**) or overexpression cells (**G**) was evaluated by western blot.

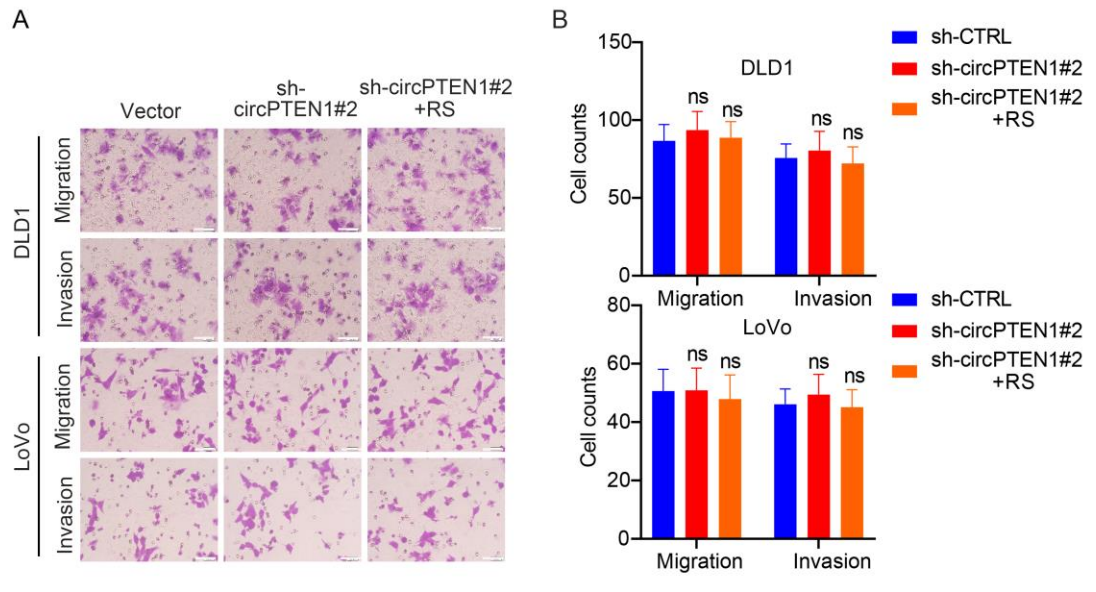

Supplementary Figure 7. The effect of circPTEN1 level on cell motility without treatment.

The motility of circPTEN1 knockdown or overexpressing cells was sequentially

evaluated through Transwell migration assays and invasion assays. (**A**) Representative images. Scale bar, 40 μm. (**B**) Migrated cells were counted in five random fields per well to calculate cell migration and invasion ability. ns: no statistically significant. RS: pLCDH-circPTEN1-RS.

A

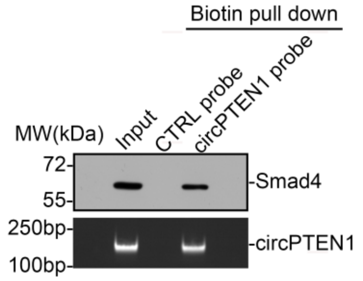

B

Accesion number	Gene	Coverage(%)	Biotin-sense Area	Biotin-antisense Area
K7EIU8	SMAD4	51.1	0	1.95E+08
P13489	RHN1	54.01	0	2.63E+07
P78527	PRKDC	9.59	0	1.02E+07
P42167	TMPO	48.46	0	5.52E+06
P42166	TMPO	43.8	0	5.52E+06
P04837	SSBP1	55.41	0	2.23E+06
P50402	EMD	37.8	0	1.79E+06
Q9UJZ1	STMOL2	41.85	0	1.38E+06
P22061	PCMT1	31.28	0	1.25E+06
P09874	PARP1	32.54	0	1.02E+06

Supplementary Figure 8. Smad4 was the protein associated with circPTEN1 with the highest abundance.

A. The Smad4 protein interacting with the circPTEN1 probe was detected by western blot.

B. The proteins associated with circPTEN1 as revealed by MS were sorted from up to low according to the coverage area.

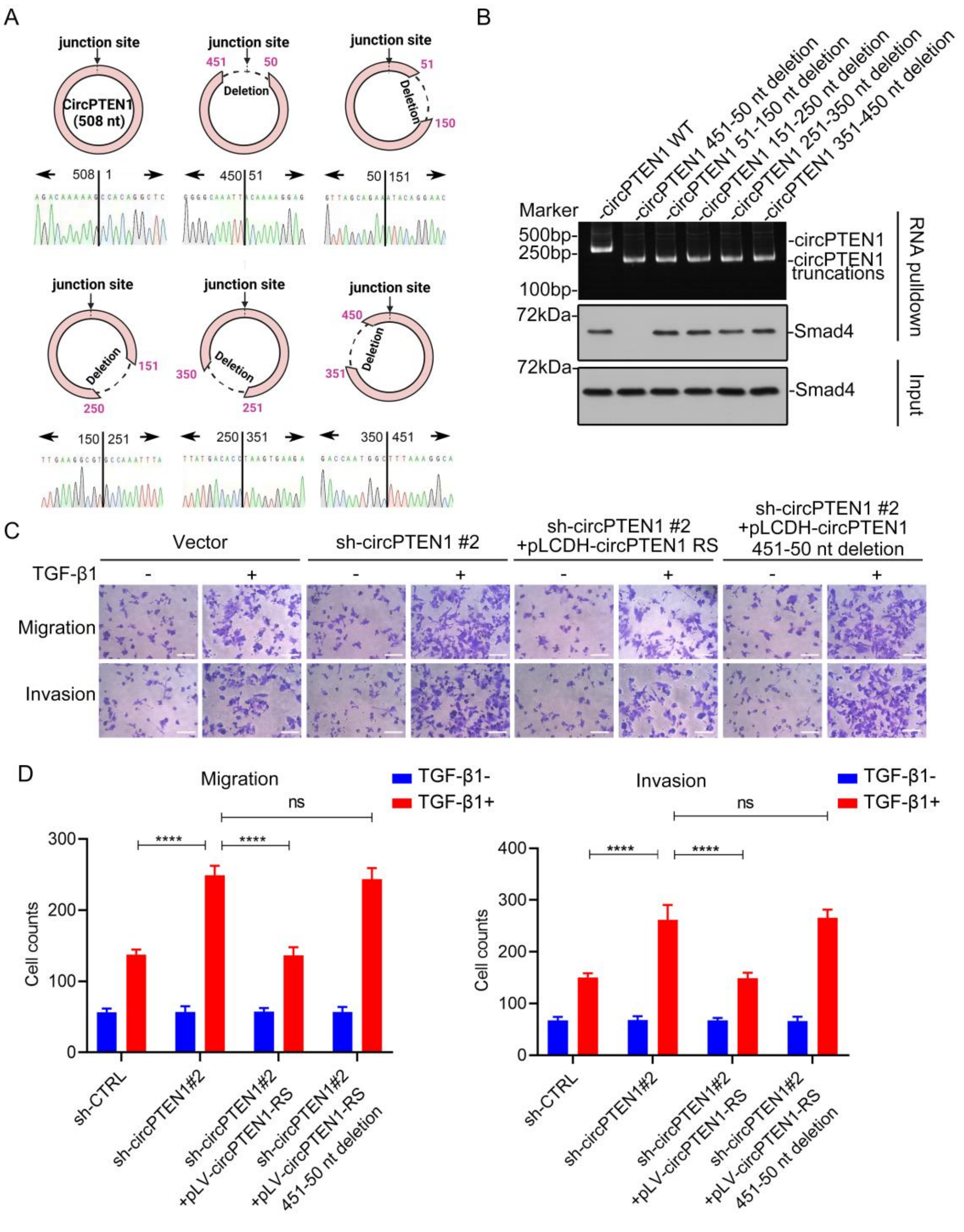

Supplementary Figure 9. The effect of circPTEN1 on the migration and invasion activities of CRC mediated by TGF-β was dependent on its interaction with Smad4.

A. A set of different circPTEN1 truncations was constructed, and these circPTEN1 truncations were introduced into LoVo cells. The enriched full-length circPTEN1 and

circPTEN1 truncations by RNA pull-down were subsequently validated by Sanger sequencing of the RT-PCR products.

B. The RNA pull-down assay was performed to analyze the interaction between Smad4 and circPTEN1 truncations.

C and **D.** The indicated LoVo cells were treated with 5 ng/mL TGF-β1 for 48 h, and motility was sequentially evaluated through Transwell migration assays and invasion assays. (**C**) Representative images. Scale bar, 40 μm. (**D**) Migrated cells were counted in five random fields per well. RS: pLCDH-circPTEN1-RS. ****, p<0.0001. ns: no statistically significance.

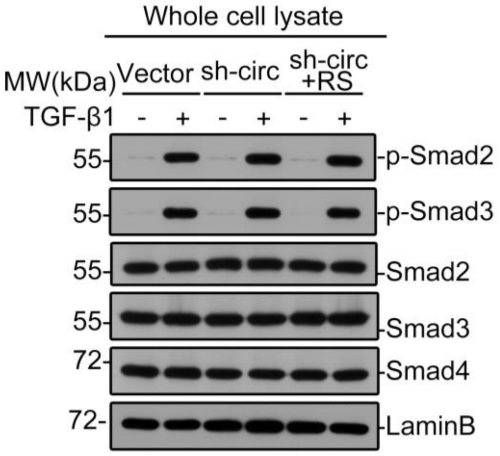

Supplementary Figure 10.The expression of p-Smad2 and p-Smad3 in whole cell lysates of the indicated cells.

The circPTEN1 knockdown cells as indicated in Supplementary Fig. 6C, and the circPTEN1 overexpression cells as indicated in Supplementary Fig. 6E were treated with 5 ng/mL TGF-β1 for 1 h. Whole cell lysates were extracted to detect the expression of the indicated proteins. RS: pLCDH-circPTEN1-RS.

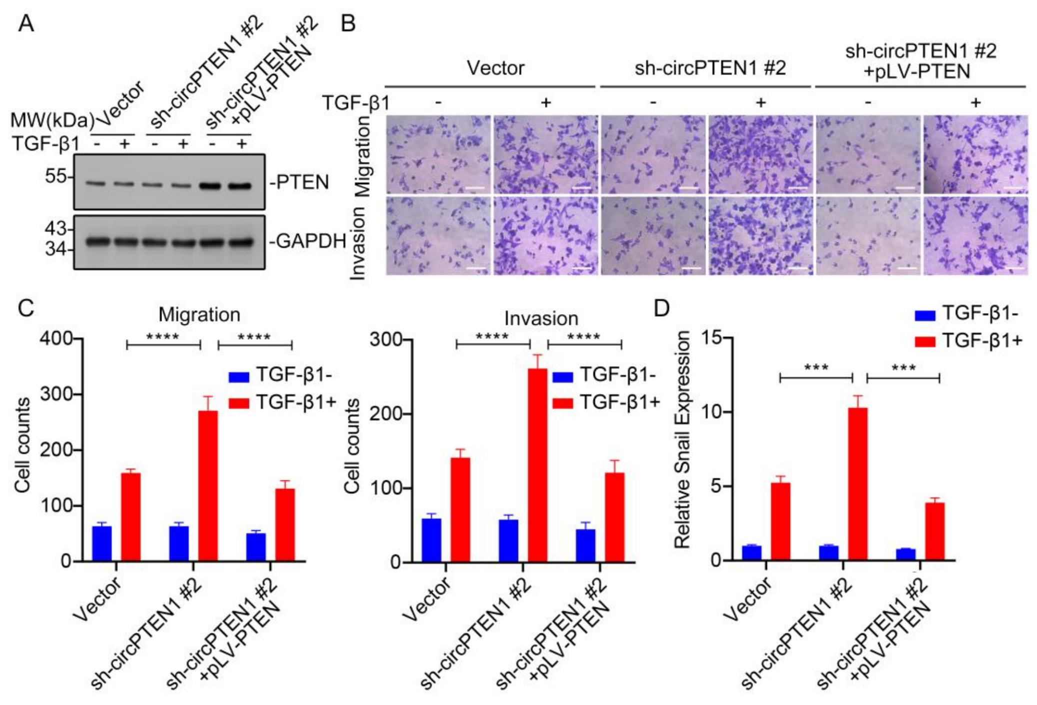

Supplementary Figure 11. The effect of overexpressed PTEN on the invasiveness of circPTEN1 knockdown LoVo cells.

A. The expression of PTEN in the indicated LoVo cells was evaluated by western blot.

B and **C.** The indicated LoVo cells were treated with 5 ng/mL TGF-β1 for 48 h, and motility was sequentially evaluated through Transwell migration assays and invasion assays. (**B**) Representative images. Scale bar, 40 μm. (**C**) Migrated cells were counted in five random fields per well. ****, $p<0.0001$.

D. The expression of Snail in the indicated LoVo cells was evaluated by qRT-PCR. ***, $p<0.001$.

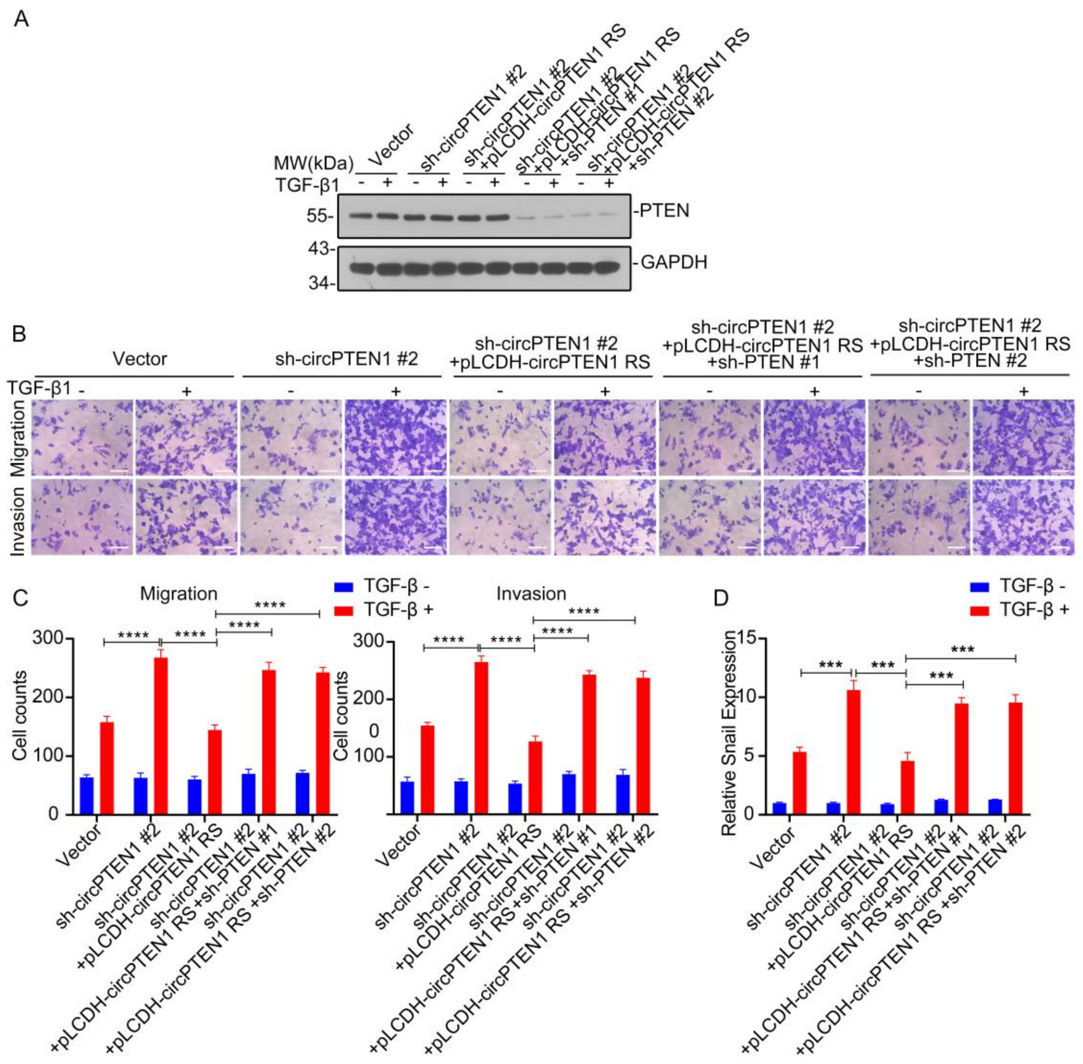

Supplementary Figure 12. The effect of PTEN knockdown on the invasiveness of LoVo cells overexpressing circPTEN1.

A. The expression of PTEN in the indicated LoVo cells was evaluated by western blot.

B and **C.** The indicated LoVo cells were treated with 5 ng/mL TGF-β1 for 48 h, and motility was sequentially evaluated through Transwell migration assays and invasion assays. (**B**) Representative images. Scale bar, 40 μm. (**C**) Migrated cells were counted in five random fields per well. ****, p<0.0001.

D. The expression of Snail in the indicated LoVo cells was evaluated by qRT-PCR. ***, p<0.001.

Supplementary Table 1. A list of antibodies used in this study

Species	Antigen	Clone #	Company (Cat#)	Dilution ratio			Citation
				Immuno blotting	IP/RIP	Immuno fluorescence	
Rabbit polyclonal	Smad2 Phospho (S465, S467)		ThemoFisher (#44-244G)	1:1000			(*3*)
Rabbit polyclonal	Smad3 Phospho (S423, S425)		ThemoFisher (#44-246G)	1:1000			(*4*)
Rabbit monoclonal	Smad2	EP784Y	Abcam (ab40855)	1:1000			(*5*)
Rabbit monoclonal	Smad3	EP568Y	Abcam (ab40854)	1:1000			(*6*)
Rabbit monoclonal	Smad4	EPR22589-112	Abcam (ab230815)	1:1000	1:200	1:100	(*7*)
Rabbit Polyclonal	Ski		Beyotime(AF7988)	1:500			N/A
Mouse monoclonal	E-cadherin	4A2	Cell Signaling (#14472)	1:1000			(*8*)
Mouse monoclonal	N-cadherin	13A9	Cell Signaling (#14215)	1:1000			(*9*)
Mouse monoclonal	Vimentin	V-9	Santa Cruz (sc-6260)	1:1000			(*10*)
Rabbit polyclonal	eIF4A3		Abcam (ab32485)	1:1000	1:200		(*11*)
Mouse monoclonal	GAPDH	1C4	Sungene Biotech (KM9002)	1:5000			N/A
Mouse monoclonal	FLAG	M2	Sigma-Aldrich (F3165)	1:5000	1:500	1:300	(*12*)

Supplementary Table 2. Oligonucleotides sequences

shRNA	Sequence(5'-3')
sh-circPTEN1#1	GACCAGAGACAAAAAGCCACA
sh-circPTEN1#2	AGAGACAAAAAGCCACAGGCT

sh-circPTEN1#3	AAAAAGCCACAGGCTCCCAGA	
sh-eIF4A3#1	GCCACCACCTTCTCTAGTAAC	
sh-eIF4A3#2	GGTCTGTCACTCATGGGTTTA	
sh-PTEN#1	GCAGTAAACTTTCAATGCTGC	
sh-PTEN#2	GGGCCAGGTCATAAATAATGA	
**Biotin labelled probes**		
**Name**	**Sequence(5'-3')**	
Detection of full length circPTEN1	TCATGTCTGGGAGCCTGTGGCTTTTTGTCTCTGGTCCT-Biotin	
Negative control probe	AGGACCAGAGACAAAAAGCCACAGGCTCCCAGACATGA-Biotin	
Detection of 451-50 nt deletion of circPTEN1	CTCTTGATATCTCCTTTTGTAATTTGCCCCGATGTAATAAATATGC-Biotin	
Detection of 51-150 nt deletion of circPTEN1	CATCATCAATATTGTTCCTGTATTTCTGCTAACGATCTCTTTGATG-Biotin	
Detection of 151-250 nt deletion of circPTEN1	CAACTCTGCAATTAAATTTGGCACGCCTTCAAGTCTTTCTG-Biotin	
Detection of 251-350 nt deletion of circPTEN1	CAACATGATTGTCATCTTCACTTAGGTGTCATAATGTCTTTCAGC-Biotin	
Detection of 351-450 nt deletion of circPTEN1	GGCCTCTTGTGCCTTTAAAGCCATTGGTCAAGATCTTC-Biotin	
**Probes for FISH**		
circPTEN1 probe	TCATGTCTGGGAGCCTGTGGCTTTTTGTCTCTGGTCCTTA-CY3	
circPTEN2 probe	CTTTTTTAGCATCTTGTTCTCATAATGTCTTTCAGCACAA-CY3	
Negative control probe	TAAGGACCAGAGACAAAAAGCCACAGGCTCCCAGACATGA-CY3	
**Primers for *in vitro* transcription**		
**Name**	**Forward (5'-3')**	**Backward (5'-3')**
T7-circPTEN1-sense (for in vitro cyclization)	taatacgactcactataGCCACAGGCTCCCAGACATG	TTTTTGTCTCTGGTCCTTACTTCC
T7-circPTEN1-antisense (for in vitro cyclization)	taatacgactcactataGGCTTTTTGTCTCTGGTCCTTAC	ACAGGCTCCCAGACATGAC

T7-circPTEN1-probes (for Northern blot)	taatacgactcactatagggGATAAGTTCTAGCTGTGGTGGG	CTGTAAAGCTGGAAAGGGACG
T7-PTEN-probes (for Northern blot)	taatacgactcactatagggTCTAGGGCCTCTTGTGCC	GCTATGGGATTTCCTGCAG
T7-circPTEN1-upstream Sense (0 ~ -1000 bp) (for Pulldown)	taatacgactcactatagggGCGGTCCCGTCCGCCTCT	CTGAAGAAAAAGGAGGAGAGAG
T7-circPTEN1-upstream antisense (0 ~ -1000 bp) (for Pulldown)	taatacgactcactatagggCTGAAGAAAAAGGAGGAGAGAGATG	GCGGTCCCGTCCGCCTCT
T7-circPTEN1-downstream sense (0 ~ 1000 bp) (for Pulldown)	taatacgactcactatagggGTAAGTTATTTTTTGATGTTTTTCCTTTC	ATGAGTTTTTCTATCTATCTGGAGG
T7-circPTEN1-downstream antisense (0 ~ 1000 bp) (for Pulldown)	taatacgactcactatagggATGAGTTTTTCTATCTATCTGGAGG	GTAAGTTATTTTTTGATGTTTTTCCTTTC
T7-circPTEN1 upstream-a1 (0 ~ -1000 bp) (for Pulldown)	taatacgactcactatagggGCGGTCCCGTCCGCCTCT	CTGAAGAAAAAGGAGGAGAGAG
T7-circPTEN1 upstream -a2 (0 ~ -700 bp) (for Pulldown)	taatacgactcactatagggGTGAGCAGCCGCGGGGGCA	CTGAAGAAAAAGGAGGAGAGAG
T7-circPTEN1 upstream -a3 (0 ~ -500 bp) (for Pulldown)	taatacgactcactatagggAGCGGGGGGGAGAAGCGG	CTGAAGAAAAAGGAGGAGAGAG
T7-circPTEN1 upstream-a4 (0 ~ -400 bp) (for Pulldown)	taatacgactcactatagggAGAGTTGGTCTCTCCCCTTCT	CTGAAGAAAAAGGAGGAGAGAG
T7-circPTEN1 upstream -a5 (0 ~ -300 bp) (for Pulldown)	taatacgactcactatagggCGCACCCCCCGTGGCCCG	CTGAAGAAAAAGGAGGAGAGAG
T7-circPTEN1 upstream -a6 (0 ~ -200 bp) (for Pulldown)	taatacgactcactatagggCTGGCTGCTGAGGAGAAGC	CTGAAGAAAAAGGAGGAGAGAG
T7-circPTEN1 downstream -a7 (0 ~1000 bp) (for Pulldown)	taatacgactcactatagggGTAAGTTATTTTTTGATGTTTTTCC	ATGAGTTTTTCTATCTATCTGG
T7-H19 (0 ~2362 bp)-probes (for Pulldown)	taatacgactcactatagggAGTTAGAAAAAGCCCGGGCTAG	GCTGTAACAGTGTTTATTGATGATG
**Primers used in identification of circRNAs**		

Name	Forward (5’-3’)	Backward (5’-3’)
hsa_circ_0002232 divergent	ACTGTAAAGCTGGAAAGGGACG	GCTAACGATCTCTTTGATGATGGC
hsa_circ_0002232 convergent	AAGACCATAACCCACCACAGC	ACCAGTTCGTCCCTTTCCAG
hsa_circ_0002934 divergent	GTAAGGACCAGAGACAAAAAGGTT	TGGTGGGTTATGGTCTTCAAAA
hsa_circ_0019058 divergent	GAAGGCGTATACAGGAACAATATTG	CCATAGCAATAATGTTTGGATAAATATC
hsa_circ_0019059 divergent	TGGCGGAACTTGCATTGTG	AGTTCTAGCTGTGGTGGGTT
hsa_circ_0019060 divergent	AGCCGTTACCTGTGTGTGGT	TAGCTGTGGTGGGTTATGGTC
hsa_circ_0003058 divergent	TTGAAAGCTGATAGAAGGAGT	TCACAATGATTACCTGGGCAAC
hsa_circ_0094342 divergent	ATTGCAAGCTGATGGGAACAG	TCTGCAGGAAATCCCATAGCA
hsa_circ_0094343 divergent	AACAATTTCAGCACCAATAAGTTATTG	GAATAGGACCTACTAGAGCAGC
β-actin divergent	AAAGGCGAGGCTCTGTGCT	GGGCTTACCTGTACACTGACTTGA
β-actin convergent	TTGTTACAGGAAGTCCCTTGCC	ATGCTATCACCTCCCCTGTGTG
**Primers used in qRT–PCR**		
**Name**	**Forward (5’-3’)**	**Backward (5’-3’)**
eIF4A3	TAGTGGGAAGCCAGTGGAAG	AGGCAGCACAATCCTCTCTT
U1	TCCCAGGGCGAGGCTTATCCATT	GAACGCAGTCCCCCACTACCACAAAT
β-actin	GTGGCCGAGGACTTTGAT	CCTGTAACAACGCATCTCAT
Snail	ATGGGTCGGAGCTGGATATG	GCTGTTCTCATGCCCATCTG
Slug	CCTGGTTGCTTCAAGGACAC	TGGAGCAGAGGTTGTTAGCA
ZEB1	GGAGACCTCTTGCCTGACTT	TTCACACCCTCCCATCCTTC
**Primers used in Figure 3c**		
**Name**	**Forward primer (5’-3’)**	**Backward primer (5’-3’)**

a downstream 329-522	TTCAGATCTTGTTCATCTTGTG	ACATGTGCTAGTATGATATGAAAATG
b downstream 838-1011	CTTGGGAGAACTTTTACAGTG	GAACTCTCTAAATGAGTTTTTCTATC
c upstream -191 ~ -38	GAGGAGAAGCAGGCCCAG	GGTGGCGGGGCTTCTTCTG
d upstream -224~-326	GTTTTAAACCTCCCGTGCG	AATGGGGAGAAGACGAATAATC
e upstream -335~ -411	GGGAACGCCGGAGAGTTG	GTCCCTGGATGTGCCAGC
f upstream -413~-554	TCGGGCGGGAGCCGGCTG	GCCCTGGAAATGGTGACA
g upstream -564~-708	CAGCGCCTGTGAGCAGCCG	TTCCACCTTCCCTTTCAGGA
h upstream -709~-829	GTTCTCTCCTCTCGGAAGCTGC	AGAGGGGCTCCGGGCCGC
i upstream -855~ -962	TCGGTCTTCCGAGGCGCC	AGCGCGTATCCTGCCGCA

Supplementary References

1. D. S. Chandrashekar *et al.*, UALCAN: A Portal for Facilitating Tumor Subgroup Gene Expression and Survival Analyses. *Neoplasia* **19**, 649-658 (2017).
2. Z. Tang *et al.*, GEPIA: a web server for cancer and normal gene expression profiling and interactive analyses. *Nucleic Acids Res* **45**, W98-W102 (2017).
3. N. V. Margaryan *et al.*, Melanocytes Affect Nodal Expression and Signaling in Melanoma Cells: A Lesson from Pediatric Large Congenital Melanocytic Nevi. *Int J Mol Sci* **17**, 418 (2016).
4. Y. Zhu *et al.*, Restenosis Inhibition and Re-differentiation of TGFbeta/Smad3-activated Smooth Muscle Cells by Resveratrol. *Sci Rep* **7**, 41916 (2017).
5. Z. Xiao *et al.*, RUNX3 inhibits the invasion and migration of esophageal squamous cell carcinoma by reversing the epithelialmesenchymal transition through TGFbeta/Smad signaling. *Oncol Rep* **43**, 1289-1299 (2020).
6. S. Qu, L. Yang, Z. Liu, MicroRNA-194 reduces inflammatory response and human dermal microvascular endothelial cells permeability through suppression of TGF-beta/SMAD pathway by inhibiting THBS1 in chronic idiopathic urticaria. *J Cell Biochem* **121**, 111-124 (2020).
7. C. Wang *et al.*, Cryptotanshinone Attenuates Airway Remodeling by Inhibiting Crosstalk Between Tumor Necrosis Factor-Like Weak Inducer of Apoptosis and Transforming Growth Factor Beta 1 Signaling Pathways in Asthma. *Front Pharmacol* **10**, 1338 (2019).
8. F. Ma *et al.*, SKIL facilitates tumorigenesis and immune escape of NSCLC via upregulating TAZ/autophagy axis. *Cell Death Dis* **11**, 1028 (2020).
9. F. Zhou *et al.*, LINC00355:8 promotes cell proliferation and migration with invasion via the MiR-6777-3p/Wnt10b axis in Hepatocellular Carcinoma. *J Cancer* **11**, 5641-5655 (2020).
10. A. Maia *et al.*, IFNbeta1 secreted by breast cancer cells undergoing chemotherapy reprograms stromal fibroblasts to support tumour growth after treatment. *Mol Oncol* **15**, 1308-1329 (2021).
11. C. Van Rechem *et al.*, Lysine demethylase KDM4A associates with translation machinery and

regulates protein synthesis. *Cancer Discov* **5**, 255-263 (2015).
12. X. Gu *et al.*, GSG1L suppresses AMPA receptor-mediated synaptic transmission and uniquely modulates AMPA receptor kinetics in hippocampal neurons. *Nat Commun* **7**, 10873 (2016).
